# Cross-cohort genetic risk prediction for Alzheimer’s disease: a transfer learning approach using GWAS and deep learning models

**DOI:** 10.1186/s13040-025-00506-0

**Published:** 2025-12-22

**Authors:** Isibor Kennedy Ihianle, Wathsala Samarasekara, Keeley Brookes, Pedro Machado

**Affiliations:** Nottingham Trent University, Nottingham, NG11 8NS UK

**Keywords:** Alzheimer’s disease, Genome-Wide association studies, Wide deep neural network, Transfer learning, Knowledge distillation, Machine learning, Risk prediction models

## Abstract

Alzheimer’s Disease (AD) represents a growing global health challenge, driven by complex genetic factors and diverse risk contributors. Currently, an estimated 55 million people worldwide are affected by dementia, with AD responsible for 60–70% of these cases. This paper explores the application of advanced machine learning approaches to predict AD risk using Genome-Wide Association Studies data from multiple cohorts, with a particular focus on transfer learning and feature selection techniques. We evaluate the performance of Wide and Deep Neural Networks and Multi-Head Attention in assessing their ability to generalise across datasets. As part of this, we explore knowledge distillation as a strategy to enhance model efficiency through improved generalisation performance in smaller architectures by transferring knowledge from high-capacity models to lightweight ones. Furthermore, the performance of these deep learning approaches is compared with tree-based ensembles, including Random Forest and XGBoost. Our experiments evaluate the generalisability, transferability, and efficiency of these models across different transfer learning scenarios. Findings indicate that aggregating multi-cohort training data significantly enhances predictive performance, highlighting the importance of data diversity in improving AD risk assessment. The proposed knowledge distillation approach enables the transfer of knowledge from a complex teacher model to a simpler student model, significantly improving performance. To enhance interpretability, we apply SHAP (SHapley Additive exPlanations) to the student models, revealing cohort-specific differences in SNP importance and highlighting variants in genes such as ABI3BP and SYN3, both of which are linked to immune and synaptic functions in AD. The integration of SHAP enables transparent interpretation of model decisions and supports the identification of transferable genetic markers, reinforcing the clinical relevance of our framework in AD risk prediction.

## Introduction

Alzheimer’s disease (AD) is a neurodegenerative disorder characterised by progressive memory loss, cognitive decline, and behavioural changes. It is the most common form of dementia, with approximately 50–70% of all dementia cases attributed to AD [1, 2]. The global burden of AD is steadily increasing, with an estimated 55 million people worldwide affected by dementia, and AD accounting for 60–70% of these cases [3]. As the population ages, the prevalence of AD is projected to rise sharply, potentially affecting 139 million individuals globally by 2050 [4].

AD is commonly classified into Early Onset Alzheimer’s Disease (EOAD) and Late Onset Alzheimer’s Disease (LOAD) forms. While EOAD, which accounts for 5–10% of cases, is often associated with deterministic mutations in genes such as Amyloid Precursor Protein (APP), Presenilin 1 (PSEN1), and Presenilin 2 (PSEN2) [5, 6], LOAD has a more complex genetic architecture involving multiple risk alleles, environmental exposures, and lifestyle factors [7, 8]. Among the genetic contributors, the Apolipoprotein E (APOE) gene, specifically the $\varepsilon4$ allele, is the most well-established risk factor for AD, associated with both increased susceptibility and earlier disease onset [9].

Beyond APOE, Genome-Wide Association Studies (GWAS) have identified over 40 additional risk loci involved in neuroinflammation, lipid metabolism, and synaptic function [10, 11]. These include variants in genes such as TREM2 and CLU, among others [12, 13]. However, the identified variants explain only a fraction of AD heritability, which is estimated to be between 58 and 79% [9]. The missing heritability and population-specific allele distributions underscore the need for advanced analytical methods that can model polygenic and cohort-specific risk patterns.

Recent advances in Machine Learning (ML) and Transfer Learning (TL) present significant opportunities for addressing the challenges of GWAS-based prediction. These approaches are capable of revealing non-linear interactions among SNPs and improving the portability of predictive models across populations. TL in particular enables models trained on one dataset to adapt effectively to genetically diverse target cohorts, thereby improving generalisability in multi-cohort settings.

In this paper, we propose a novel cross-cohort transfer learning framework using Knowledge Distillation (KD) to enhance AD risk prediction from GWAS data. Unlike previous works using standard classifiers or deep models within a single cohort, our approach uniquely integrates KD to compress knowledge from a deep teacher model into a lightweight student model. We demonstrate that the student model trained via KD not only generalises better than its teacher but also supports downstream classification tasks using traditional models such as Random Forest (RF) and XGBoost. Features extracted from the fine-tuned teacher network serve as a biologically informed representation of the genetic signal, allowing tree-based models to outperform deep networks in several cases, particularly when trained on aggregated or cross-cohort data. To enhance model transparency and address the interpretability gap often associated with deep learning in genomics, we further integrate SHAP (SHapley Additive exPlanations) analysis into our pipeline [14]. SHAP is applied to the student models to quantify SNP level importance across target cohorts, providing interpretable insight into how transferred knowledge is reweighted by the student models in different genomic contexts. Unlike many existing studies that prioritise predictive performance alone, our work contributes to biological interpretability by identifying both known AD-associated variants and potential novel cohort-specific signals. This makes the framework not only predictive but also biologically informative, a key distinction from previous cross-cohort GWAS studies. In summary, this paper makes the following novel contributions to the field of AD risk prediction:

- Introduction of a cross-cohort KD framework for AD risk prediction using GWAS data, demonstrating improved generalisability and efficiency across genetically diverse cohorts.
- Use of teacher extracted features to enhance the performance of traditional classifiers, including RF„ XGBoost, demonstrating that tree-based models can outperform deep networks when informed by biologically meaningful representations.
- Systematic benchmarking across multiple cohort combinations to evaluate model robustness, demonstrating that aggregated training data improves both accuracy and interpretability.
- Integration of SHAP analysis to interpret model behaviour at the SNP level, enhancing biological transparency and identifying transferable versus cohort-specific variants.

The remainder of this paper is structured as follows. The related work is presented and analysed in Sect. Related work, and the proposed methodology is discussed in Sect. Proposed methodology. The results and discussion are presented in Sect. Dataset, Limitations, Ethical Considerations, and Practical Implications in Sect. Association test and the conclusions and future work are discussed in Sect. Feature selection.

## Related work

GWAS has been instrumental in identifying genetic variants associated with AD. Large-scale studies have revealed critical susceptibility loci, with the APOE gene emerging as the most significant contributor to AD risk [15] by analysing Single Nucleotide Polymorphism (SNP)s across the genome. The APOE $\varepsilon4$ allele is the strongest known genetic risk factor for late-onset AD, whereas the $\varepsilon2$ allele is considered protective. In addition to APOE, meta-analyses have identified numerous other loci implicated in key biological pathways such as amyloid-*β* processing, tau pathology, neuroinflammation, and lipid metabolism [16, 17]. These confirm the polygenic nature of AD and highlight the importance of genetic diversity in risk prediction frameworks.

However, many GWAS findings suffer from limited generalisability due to cohort-specific biases. Variations in sample size, ancestry, imputation pipelines, and genotyping arrays lead to inconsistencies in SNP effect sizes and model portability [18]. A large proportion of GWAS have focused on European-ancestry populations, with significant underrepresentation of African, Hispanic, and Asian cohorts [19], limiting the applicability of existing models to diverse populations. While some multi-cohort or meta-analytic studies aim to address this by increasing statistical power [20], they often overlook non-linear dependencies, cohort-specific feature interactions, and do not adaptively learn across heterogeneous populations. Therefore, novel frameworks are required that go beyond aggregation to support true knowledge transfer across cohorts while preserving interpretability.

Feature selection plays a key role in addressing GWAS-specific challenges such as high dimensionality and feature redundancy [21]. Traditional methods like logistic regression, chi-square tests, and Principal Component Analysis (PCA) have been widely applied. More recently, machine learning-based approaches such as mutual information (MI), F-score, Boruta, and ensemble methods like random forests and gradient boosting have demonstrated improved SNP prioritisation through adaptive feature ranking [22–24]. Hybrid approaches combining multiple techniques have also emerged to select biologically meaningful subsets of SNPs. Yet, most of these techniques are used within-cohort settings and do not explicitly evaluate their robustness across cohorts. Moreover, many rely purely on feature importance scores from individual algorithms, without considering how feature relevance shifts during transfer learning.

In this context, TL has gained traction for improving the adaptability of GWAS-based predictive models across populations. TL allows models to leverage knowledge from one cohort to inform predictions in another, mitigating data scarcity and cohort imbalance issues [25]. Recent studies have explored multi-task learning [26], domain adaptation [27], and KD-based frameworks for efficient model distillation in genomics and biomedical imaging [28, 29]. However, most of these applications either use standard neural architectures without feature interpretability or omit the role of cohort-specific reweighting in their transfer mechanisms. Importantly, none of these prior works incorporates interpretability tools such as SHAP to query model behaviour and feature salience across domains, a critical gap when aiming to build transparent, trustworthy genomic models.

Wide Deep Neural Networks (WDNN) have shown strong performance in genomic prediction tasks due to their capacity to model both low- and high-order interactions among SNPs [30]. Alatrany et al. [22] applied WDNN to within-cohort AD prediction using GWAS data, demonstrating competitive classification performance. However, their work was limited to single-cohort analysis and did not consider cross-cohort transferability, nor did it incorporate any mechanisms for explaining feature contributions or assessing the generalisability of extracted representations across diverse populations.

In contrast, this paper extends this direction by introducing a cross-cohort knowledge distillation framework in which WDNN serve as feature extractors, and student models are trained on genetically distinct target cohorts. Importantly, we address the interpretability gap in existing transfer learning approaches by integrating SHAP, enabling us to quantify the influence of individual SNPs on model predictions and to examine how feature importance shifts across cohorts. This provides valuable insight into which genetic signals are preserved or reweighted as knowledge is transferred from a source cohort to a new population. Furthermore, by applying both neural and traditional classifiers to the extracted features, we demonstrate that improved performance is driven not solely by model architecture, but by the quality and generalisability of the learned representation. To our knowledge, no prior work has simultaneously combined KD, WDNN, and SHAP-based interpretability for GWAS-based AD prediction across multiple cohorts. This positions our study as a novel contribution at the intersection of cross-cohort transfer learning, model interpretability, and genomic risk modelling.

## Proposed methodology

**Fig. 1:**
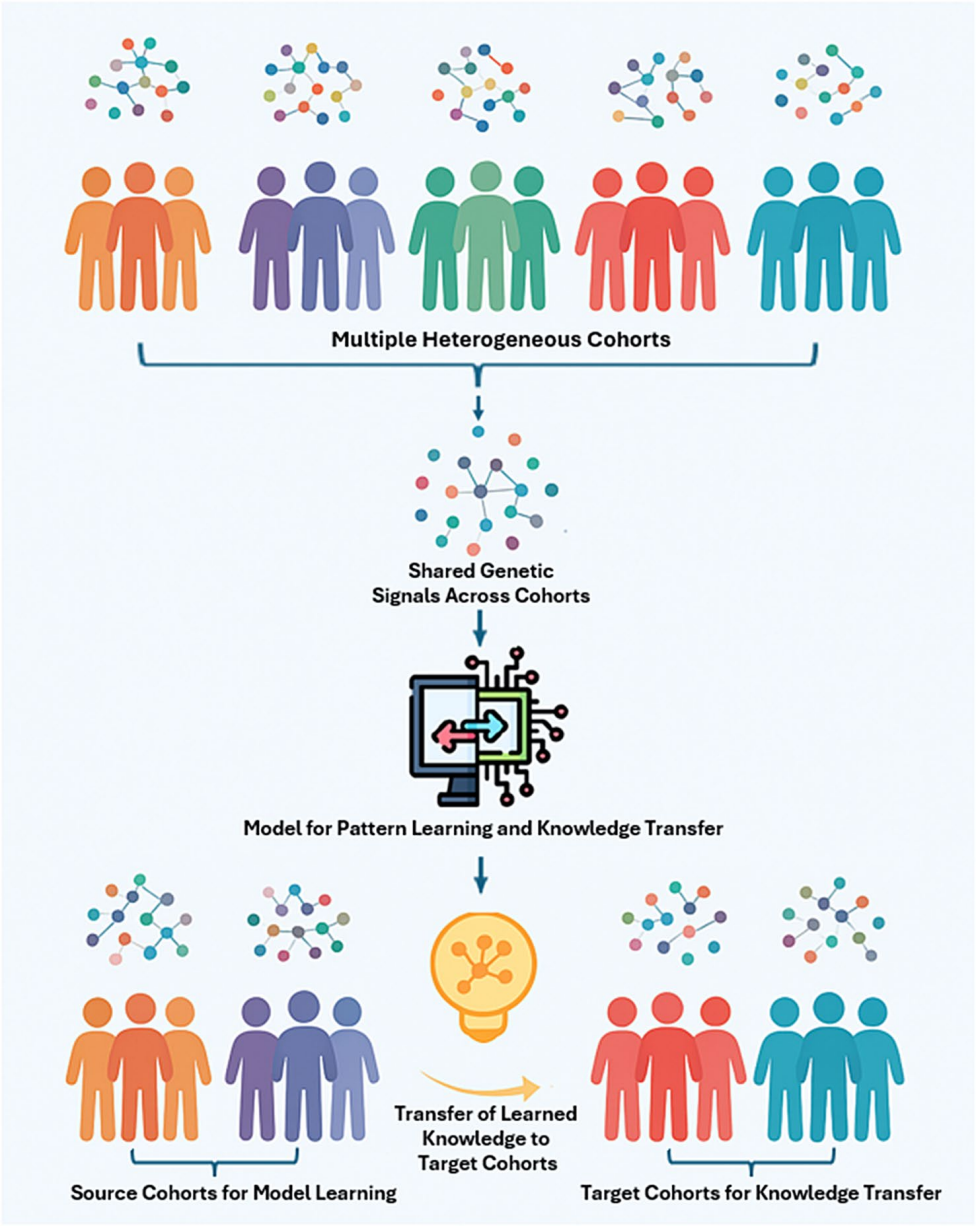
Conceptual representation of the workflow for extracting shared genetic signals fromheterogeneous cohorts and transferring learned knowledge to target cohorts

We propose a methodology leveraging transfer learning KD to enhance the cross-cohort generalisability of AD risk prediction models trained on GWAS data. To address the challenges posed by the high dimensionality and sparsity inherent in GWAS datasets, we implement a feature selection process to extract genetic markers with meaningful patterns. This process involves identifying and retaining the most important and common SNPs across cohorts that contribute significantly to AD risk. Our approach centres on the transfer of knowledge from a source model trained on one or multiple cohorts to a target cohort as depicted in Fig. 1. By doing so, we improved model performance across diverse populations, ensuring that the model performance is maintained or enhanced when applied to heterogeneous datasets.

### Dataset

We utilised five genotyping datasets in this study ADC7, BDR, NIA, TGEN, and ROSMAP - see the Data Availability section for details. The BDR cohort genotypes are accessible via the Dementia Platform UK^1^, with detailed information provided in Young et al. [31]. The remaining four cohorts were obtained from the National Institute on Aging Genetics of Alzheimer’s Disease Data Storage Site (NIAGADS)^2^. These datasets were selected based on their diverse genotyping platforms, varying sample sizes, and availability of APOE isoform/genotype data. The ADC7 dataset represents the seventh set of genotyped subjects from the Alzheimer’s Disease Centers, used by the Alzheimer’s Disease Genetics Consortium (ADGC) to identify AD-associated genes [16]. The ROSMAP (ROS) cohort provides a comprehensive multi-omic atlas of the human frontal cortex, facilitating ageing and AD research [32]. Although these data sets offer valuable genetic information, some established AD-associated genes, including the APOE SNPs rs429358, rs7412 and many others are notably absent from the ROS cohort. This discrepancy, coupled with the variability and diversity of SNPs across the datasets, highlights a critical challenge in cross-cohort analysis, which our work addresses through the application of common feature selection and transfer learning techniques. It also reflects real-world variability in genetic associations, highlighting the importance of methods that generalise across cohort-specific genetic architectures. Table 1 provides an overview of the dataset and the number of AD and Control samples in each cohort.

**Table 1:** Number of AD and control samples per cohort

Cohort	AD	Control	Total
ADC7	902	560	1462
BDR	359	175	534
NIA	825	561	1386
ROS	167	73	240
TGEN	751	559	1310

### Association test

**Fig. 2:**
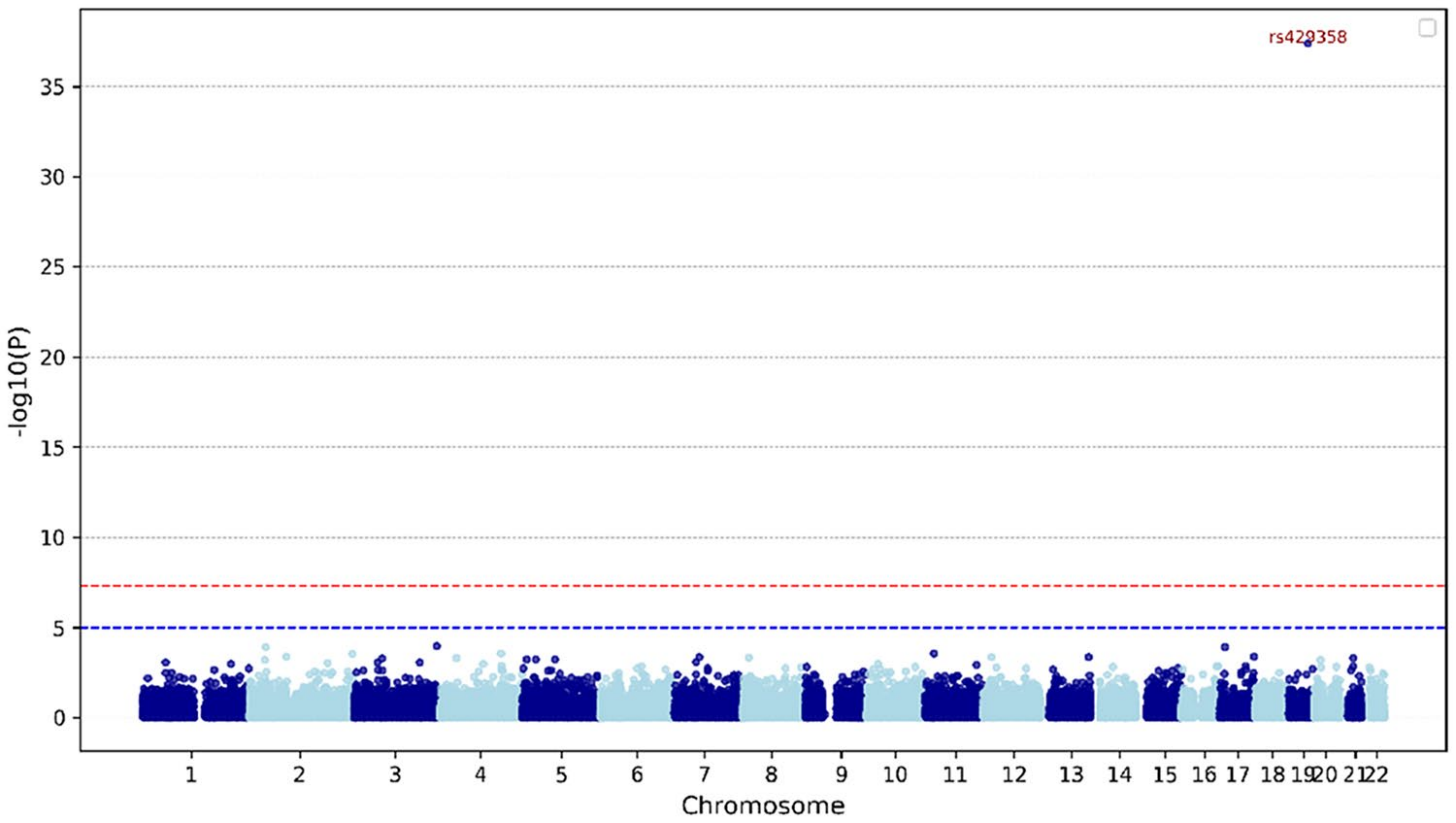
Manhattan plot of SNP associations with annotated significant SNPs for the ADC7 cohort

As part of the data processing, each cohort dataset undergoes rigorous quality control to ensure consistency across cohorts. This process includes handling missing genotype data by employing methods such as mean imputation and nearest-neighbour approaches to maintain alignment across SNPs in each dataset. Genotype counts are normalised to minimise cohort-specific biases, and feature filtering is performed based on statistical significance and biological relevance. The target variable, AD risk, is encoded consistently across datasets, with labels assigned for AD and Control cases. Figure 2 presents a sample Manhattan plot for the ADC7 cohort, illustrating the association between specific SNPs and AD risk. In this plot, genomic coordinates are displayed along the x-axis, while the negative logarithm of the association p-value for each SNP is shown on the y-axis. Notably, the SNP rs429538 is positioned above and outside the significance threshold, indicating a strong association with AD.

### Feature selection

Feature selection is a critical step in developing effecient predictive models, particularly when dealing with high-dimensional datasets such as with GWAS data. The goal of feature selection is to identify the most informative features that contribute significantly to the predictive task while reducing the dimensionality of the feature space. This reduction not only enhances computational efficiency but also mitigates the risk of redundancy and overfitting from excessive noise associated with high-dimensional data.

Numerous feature selection techniques have been proposed in the literature, including F-score methods [24, 33], Mutual Information (MI) [34], Minimum Redundancy Maximum Relevance (mRMR) [34, 35].

**Fig. 3:**
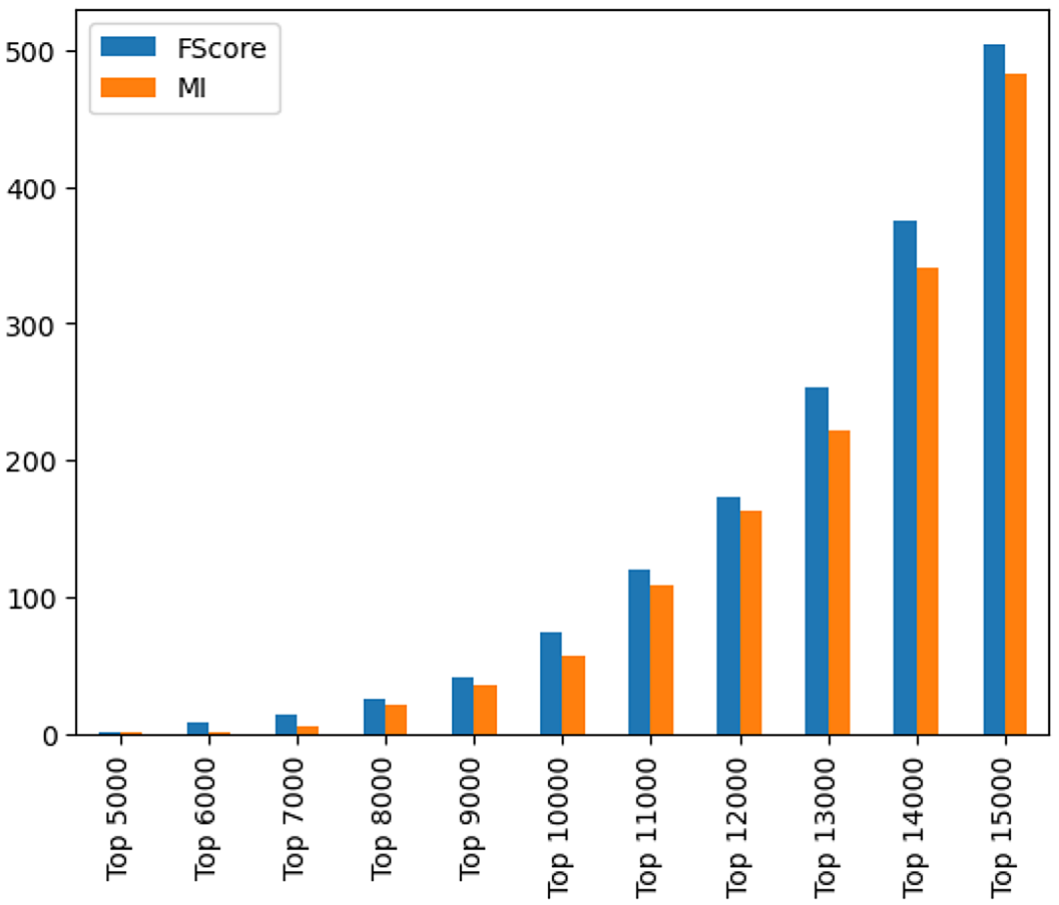
Feature comparisons of selected features across different thresholds using F-score and mutual information, highlighting their ranking differences for GWAS-based data

In this study, an F-score feature selection method is used due to its ability to effectively capture non-linear dependencies between SNPs and AD risk, aligning well with the complex genetic relationships inherent in GWAS data. From an initial set of 29,630 SNPs, the top 75 SNPs consistent and common to all the cohorts were ranked and selected based on their importance measured as the ratio of variance between classes to the variance within classes. This approach prioritised SNPs with known or hypothesised links to AD, ensuring that selected features were both biologically relevant and statistically significant. Selecting a consistent set of features across cohorts is essential for robust and unbiased cross-cohort comparisons, as it enables models to be trained on a stable set of genetic markers. To maintain consistency, we harmonised SNPs across cohorts using their respective rsIDs. Figure 3 illustrates the number of selected features at various thresholds (ranging from the top 5000 to top 15,000 features) using F-score (blue) and MI (orange). This comparison shows the differences in feature importance rankings between the two selection techniques, demonstrating their respective sensitivities to distinct SNP patterns associated with AD. The observed increase in feature overlap with expanding feature sets indicates that both methods consistently capture informative SNPs. However, discrepancies in their ranking criteria lead to variations in SNP prioritisation across selection thresholds. To establish a transferable and generalisable feature set, we ranked the features within each cohort and selected only the SNPs that consistently ranked top across ADC7, BDR, NIA, ROS, and TGEN. If an SNP was highly ranked in some cohorts but absent or ranked low in others (e.g., ROS), it was excluded from the final feature set. Without this shared feature set, cross-cohort transfer learning would be less reliable. Rather than selecting cohort-specific features, our approach focused on identifying those that were informative across all cohorts, ensuring better generalisation and robustness in cross-cohort applications. Table 2 shows the list of 75 common SNPs selected, representing the top consistent features common to all the cohorts.

**Table 2:** List of 75 common SNPs selected from ADC7, BDR, NIA, ROS and TGEN. These SNPs represent the top consistent features common to all the cohorts

1	rs10508265_A	16	rs4927219_A	31	rs12462481_A	46	rs2972174_A	61	rs13298283_C
2	rs17126761_G	17	rs4479759_G	32	rs2017392_G	47	rs8130680_G	62	rs2216982_A
3	rs2037580_G	18	rs8023522_G	33	rs1437763_G	48	rs3858961_A	63	rs698331_G
4	rs6848074_A	19	rs9815217_A	34	rs4678730_G	49	rs1447698_A	64	rs2239247_A
5	rs2029795_A	20	rs2442756_C	35	rs13387221_A	50	rs7121214_A	65	rs13360277_G
6	rs11134106_A	21	rs6840096_A	36	rs4426936_A	51	rs2958089_G	66	rs6095130_A
7	rs16893468_A	22	rs2297056_G	37	rs3813668_A	52	rs8017800_A	67	rs12308701_A
8	rs11954300_A	23	rs9582126_A	38	rs10022424_A	53	rs6880685_A	68	rs1792137_C
9	rs1345887_C	24	rs4659625_A	39	rs7433180_G	54	rs17638339_G	69	rs6460468_G
10	rs6430841_G	25	rs1893791_G	40	rs9508_G	55	rs380691_G	70	rs544500_A
11	rs31039_G	26	rs6038332_A	41	rs1407469_A	56	rs1258732_A	71	rs16965220_A
12	rs11145117_A	27	rs2242308_A	42	rs941130_A	57	rs7245541_G	72	rs984802_A
13	rs2286814_A	28	rs1736058_A	43	rs6869437_A	58	rs1879688_G	73	rs2289059_G
14	rs12736883_A	29	rs543293_A	44	rs2407211_G	59	rs1663762_A	74	rs7253210_G
15	rs9455679_A	30	rs7671299_G	45	rs2160327_G	60	rs7902395_G	75	rs2089253_G

### Proposed transfer learning technique

**Fig. 4:**
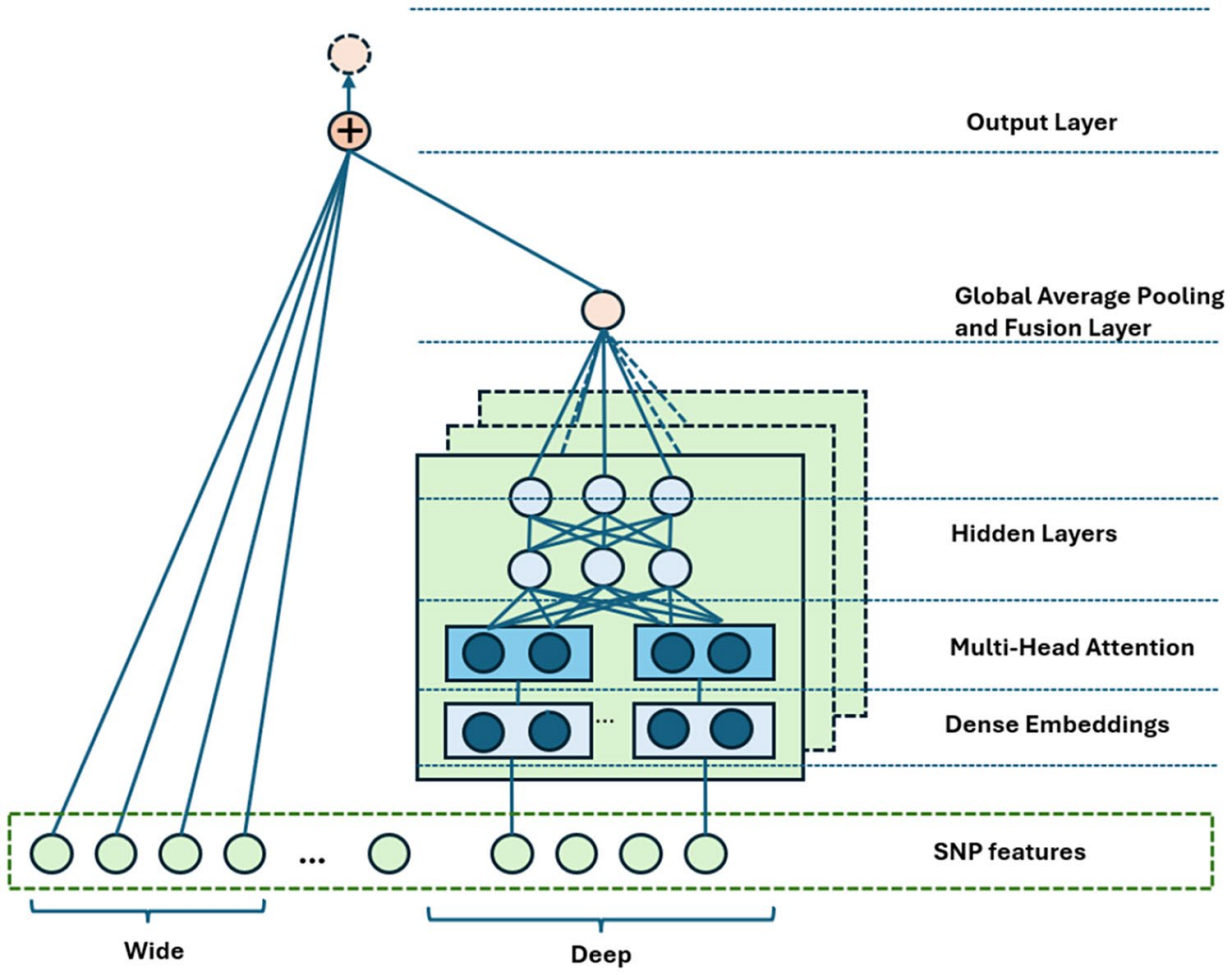
A WDNN with MHA and Fusion, representing the general structure of the proposed model. The architecture combines wide and deep components to capture both low and high-order feature interactions from SNP data. The wide component processes raw SNP features, while the deep component uses dense embeddings, MHA, and fusion layers to learn complex dependencies before prediction

We implemented a transfer learning approach designed to address the challenge of cross-cohort generalisability associated with AD risk prediction from diverse GWAS data. This approach transfers knowledge from a complex “teacher” model to a computationally efficient “student” model while retaining predictive performance [36]. This method is particularly effective for scenarios with diverse genetic and demographic contexts, ensuring adaptability across heterogeneous cohorts. For the teacher model, we utilise a WDNN model enhanced with Multi-Head Attention (MHA) mechanisms to extract patterns and significant SNPs linked to AD. The WDNN architecture, as shown in Fig. 4, integrates both a wide component for memorisation and a deep component for generalisation. The wide component consists of a single-layer perceptron that captures raw features and cross-product transformations of categorical features, enabling the model to learn linear relationships. If *x* represents the raw inputs and $\phi(x)$ their cross products, the wide model output is formulated as:

1 $$y = W_\text{wide}^T [x, \phi(x)] + b$$

where *W*_wide_ and *b* are the weights and bias of the wide network, respectively. In parallel, the deep component learns complex feature interactions through multiple hidden layers with each layer applying the following transformation:

2 $$a^{(l+1)} = f(W^{(l)}_\text{deep} a^{(l)} + b^{(l)})$$

where *f* is the activation function (e.g., ReLU), and $W^{(l)}_\text{deep}$, $a^{(l)}$, and $b^{(l)}$ are the weights, activations, and biases of the *l*-th layer. The output of the network integrates both the wide and deep components, leading to the classification probability *P*:

3 $$P(Y = 1 | x) = \sigma(W_\text{wide}^T [x, \phi(x)] + W_\text{deep}^T a^{(L)} + b)$$

where *σ* is the sigmoid activation function and $a^{(L)}$ represents the final hidden layer output. To enhance feature representation, we integrate a MHA mechanism into the deep network. The MHA allows the model to focus on different aspects of the input simultaneously, improving learning efficiency. The attention mechanism is defined as:

4 $$\text{Attention}(Q, K, V) = \text{softmax} \left(\frac{QK^T}{\sqrt{d_k}}\right) V$$

where *Q*, *K*, and *V* are the query, key, and value matrices, respectively, and *d*_*k*_ is the scaling factor. Each attention head is computed as:

5 $$\text{head}_i = \text{Attention}(QW_Q^i, K W_K^i, V W_V^i)$$

Multiple attention heads are concatenated and transformed using *W*_*O*_:

6 $$\text{MultiHead}(Q, K, V) = \text{concat}(\text{head}_1, \ldots, \text{head}_m) W_O$$

The global average pooling and fusion layer aggregates the learned representations from both the wide and deep components before passing them to the output layer. The student model is a simplified WDNN with reduced attention complexity and fewer parameters, distilling knowledge from the teacher model to balance computational efficiency and predictive accuracy. The KD process involves training the student model to replicate the behaviour of the teacher model by minimising a combined loss function. This function integrates the traditional cross-entropy loss with a distillation loss that measures the difference between the softened output probabilities (logits) of the teacher and student models. The teacher model produces a probability distribution *p*_*i*_ over classes using a softmax function with a temperature parameter *T*:

7 $$p_i = \frac{\exp\left(\frac{z_i}{T}\right)}{\sum_j \exp\left(\frac{z_j}{T}\right)}$$

where *z*_*i*_ represents the logits for class *i*, and *T* is the temperature. A higher *T* produces a softer probability distribution, generating more knowledge about the relationships between classes. The distillation loss is defined as:

8 $$L_{\text{distill}} = -\sum_i p_i^{\text{(teacher)}} \log p_i^{\text{(student)}}$$

The total loss function $\mathcal{L}$ is a weighted sum of the distillation loss and the standard cross-entropy loss with the true labels:

9 $$\mathcal{L} = \alpha L_{\text{CE}} + (1 - \alpha) L_{\text{distill}}$$

where *α* is a hyperparameter balancing the two loss components, and *L*_CE_ represents the cross-entropy loss between the student model predictions (with *T* = 1) and the true labels. The student model learns to replicate the behaviour of the teacher model by optimising this combined loss function, while also aligning with the true labels enhancing knowledge transfer.

### Experimental methodology

**Fig. 5:**
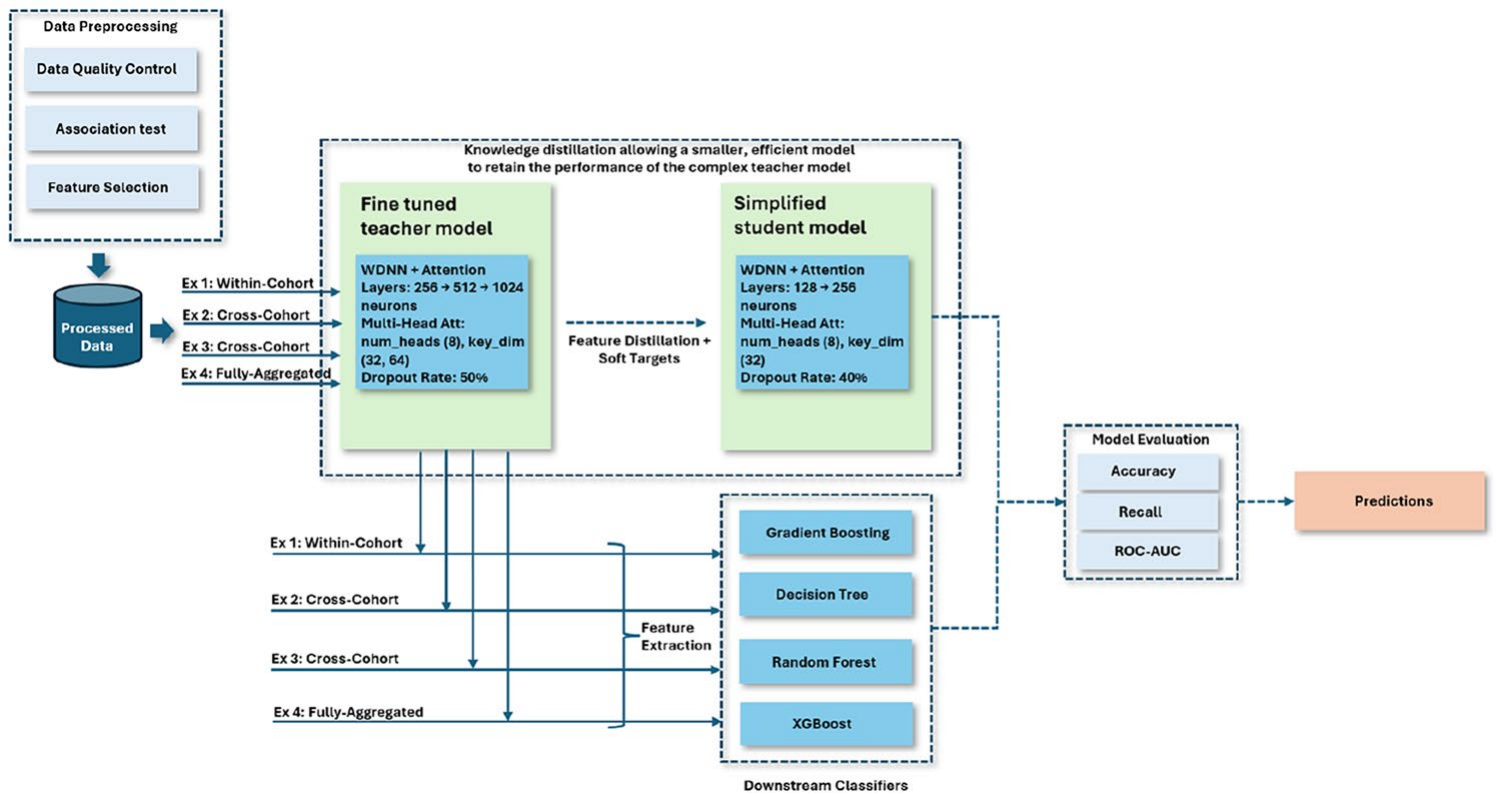
Architecture of the proposed approach illustrating the transfer learning strategy: (i) KD from the fine-tuned teacher model to a lightweight student model, and (ii) deep feature extraction from the teacher model to train traditional classifiers

To evaluate the effectiveness of the proposed KD approach in improving cross-cohort generalisability for AD risk prediction, we implemented a transfer learning pipeline combining a WDNN teacher model with a lightweight student model. The WDNN architecture was enhanced with attention mechanisms to better capture SNP interactions relevant to AD. As illustrated in Fig. 5, knowledge was transferred via two pathways: (i) distillation of soft labels from the teacher to the student model, and (ii) extraction of high-level features from the trained teacher network for training traditional classifiers such as RF, Gradient Boosting (GB), XGBoost, and Decision Tree (DT).

To mitigate the strong class imbalance typically present in GWAS datasets, we applied a two-stage balancing strategy. Random oversampling was applied to the minority class, followed by random undersampling of the majority class to ensure a balanced distribution between AD and control cases within each cohort. This process reduced model bias and improved robustness in downstream classification. The data preprocessing pipeline also included mean imputation for missing genotypes, stratified train-test splits, and the selection of common SNPs across cohorts based on both statistical relevance (F-score ranking) and biological plausibility.

Model training was implemented around a teacher-student approach. For each transfer scenario, a WDNN model was trained on the source cohort using an 80/20 stratified split. A 5-fold cross-validation was applied to the training data for hyperparameter optimisation. The student model was then trained using a composite loss function that combined soft target alignment with the teacher logits and hard-label classification using binary cross-entropy. The contribution of each loss was weighted by a tunable hyperparameter *α*, which was optimised over the range $[0.1, 1.0]$ using validation performance.

In parallel with knowledge distillation, the final trained teacher model was used as a deep feature extractor. Hidden-layer activations from the teacher were extracted for each sample and used to train traditional classifiers. This hybrid strategy allowed us to leverage the abstract representation power of the deep model while also capitalising on the interpretability and computational efficiency of tree-based learners. The overall training strategy, including feature selection, teacher training, student distillation, and feature reuse, is summarised in Algorithm 1.

**Algorithm 1:**
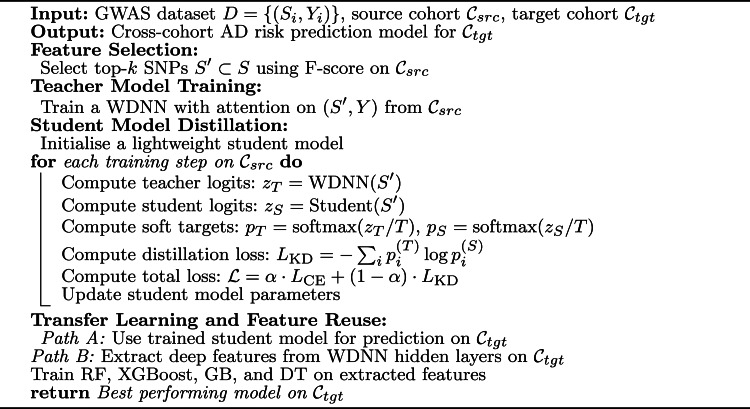
Proposed knowledge distillation-based transfer learning for GWAS

To evaluate the generalisability of the proposed framework, we designed four experiments based on different cohort configurations:

- **Experiment 1: Within-Cohort Transfer (Single Source** $\rightarrow$ **Single Target)** Assesses the ability of a model trained on a single cohort (e.g., ADC7) to generalise to others (e.g., BDR, NIA, ROS, TGEN), assuming moderate genetic overlap.
- **Experiment 2: Aggregated Source** $\rightarrow$ **Single Target** Evaluates whether training on a combined source dataset (e.g., ADC7 + BDR) enhances predictive accuracy when tested on individual target cohorts such as NIA or ROS.
- **Experiment 3: Single Source** $\rightarrow$ **Aggregated Target** Tests the generalisability of a model trained on one cohort (e.g., ADC7) when applied to a merged target cohort (e.g., BDR + NIA), simulating more heterogeneous deployment settings.
- **Experiment 4: Fully Aggregated Transfer** (**Source** $\rightarrow$ **Target)** Investigates whether training and testing on aggregated cohorts (e.g., ADC7 + BDR $\rightarrow$ NIA + ROS + TGEN) maximises model robustness across genetically diverse populations.

To address the interpretability of the KD-based predictions, we applied SHAP to each student model post-training. This was motivated by the need to understand how feature importance shifts as the model adapts to a new cohort and to examine which SNPs carry forward the knowledge encoded by the teacher. SHAP was only applied to the student models in Experiment 1, as they encapsulate the adapted knowledge specific to each target domain. This integration of SHAP enhances the biological transparency of the framework and complements the model performance analysis with mechanistic insight into SNP-level contributions.

## Results and discussion

The proposed framework was implemented in Python using established libraries for machine learning and deep learning. Scikit-learn was used to develop and evaluate traditional classifiers, while Keras with TensorFlow served as the backend for training the neural network-based models. To evaluate model performance, we used a set of metrics including accuracy, F1-score, specificity, and Area Under the Curve (AUC). Given the class imbalance often inherent in GWAS datasets, we emphasised F1-score and specificity as complementary to accuracy. The F1-score, which captures the harmonic mean of precision and recall, is particularly appropriate for evaluating imbalanced data, while specificity measures the ability to correctly identify negative (control) cases critical in clinical risk prediction settings.

### Exp 1: Within-cohort transfer (single source $\rightarrow$ single target)

**Fig. 6:**
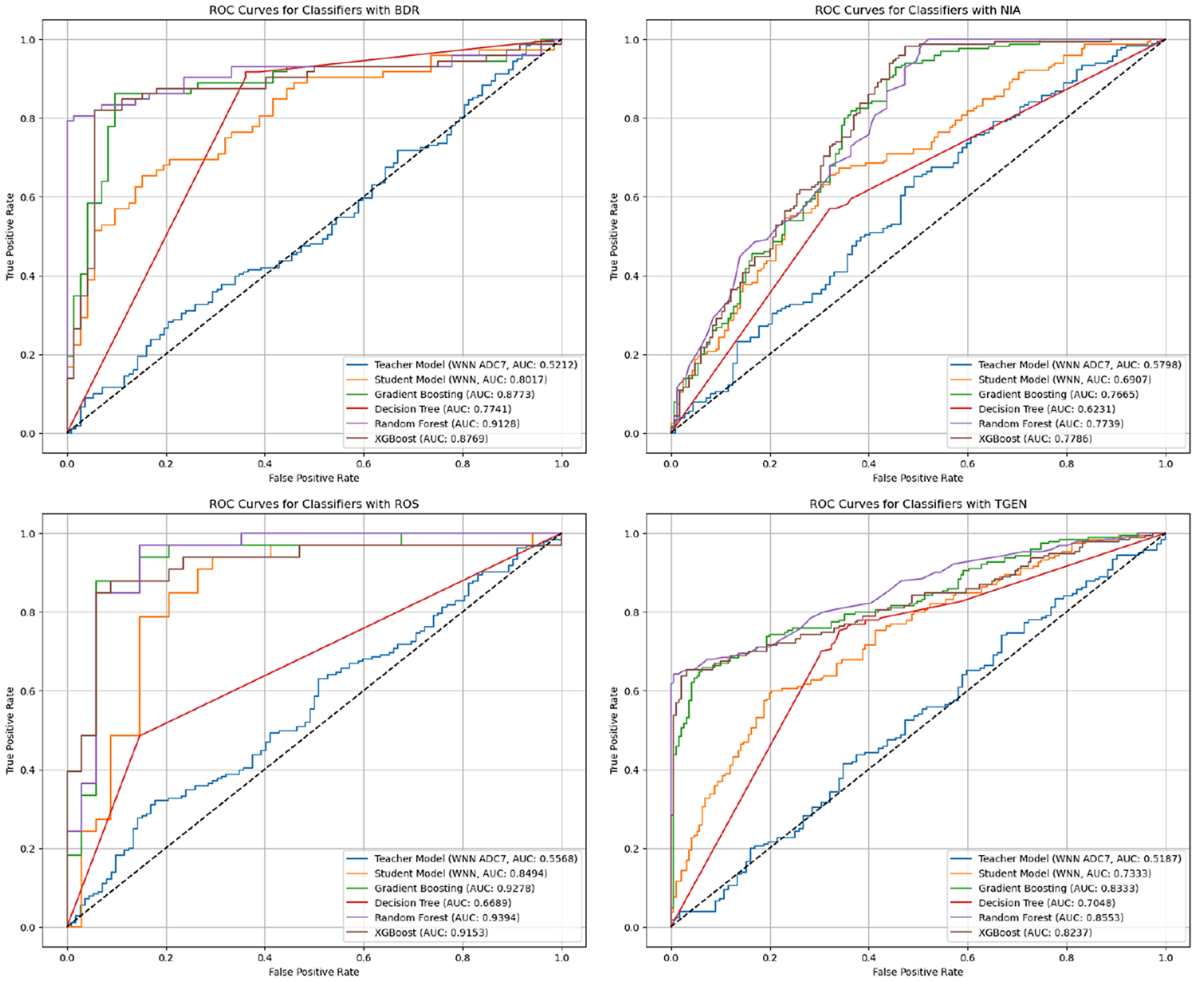
ROC curves comparing the performance of different classifiers for within-cohort transfer learning in experiment 1. The curves illustrate the trade-off between the true positive rate and false positive rate for each model across different target datasets (BDR, NIA, ROS, and TGEN), with AUC scores indicating classification effectiveness

**Fig. 7:**
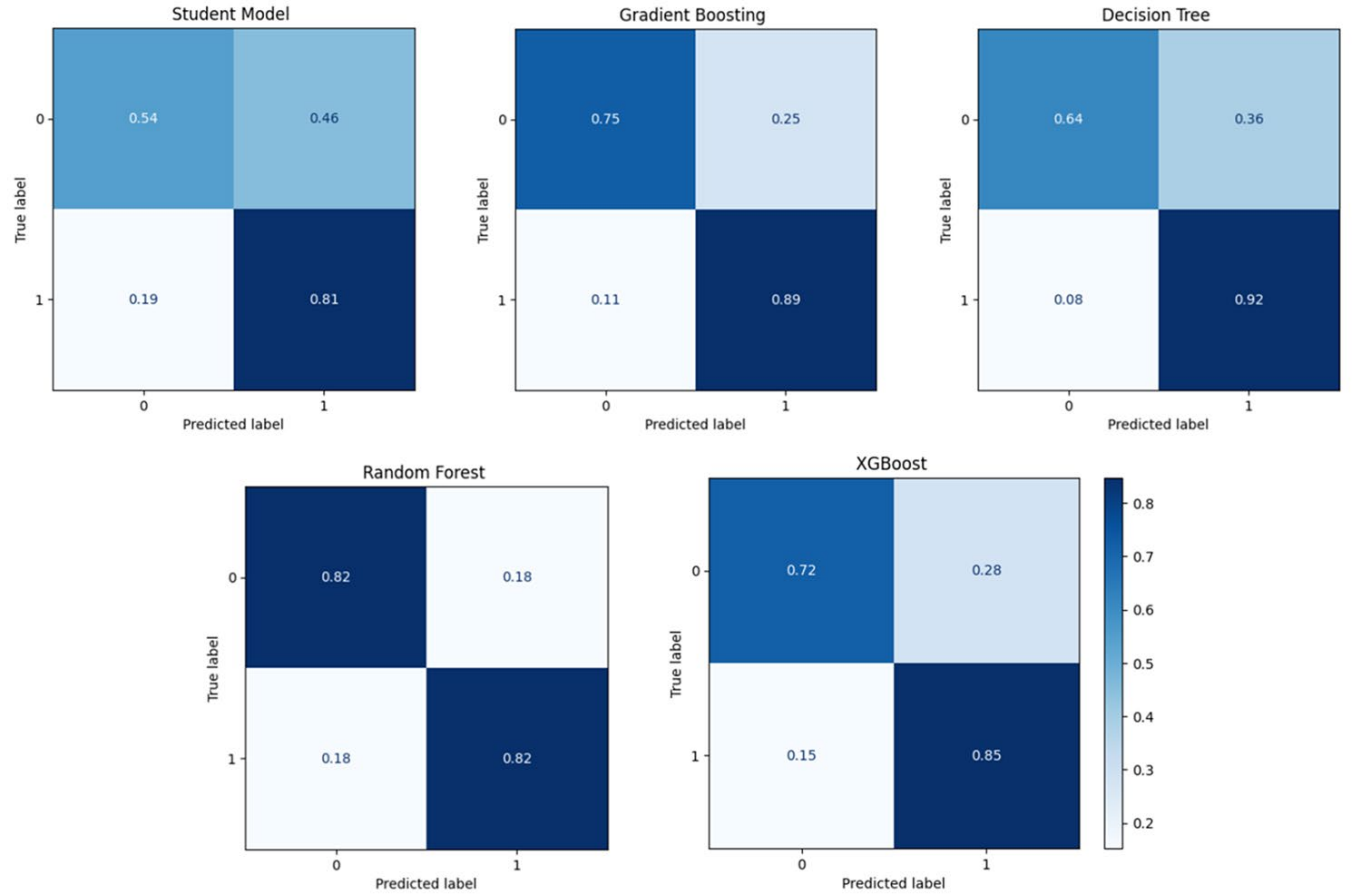
Confusion matrix for experiment 1 showing model predictions on the BDR cohort using the student model trained with knowledge distilled from ADC7

**Fig. 8:**
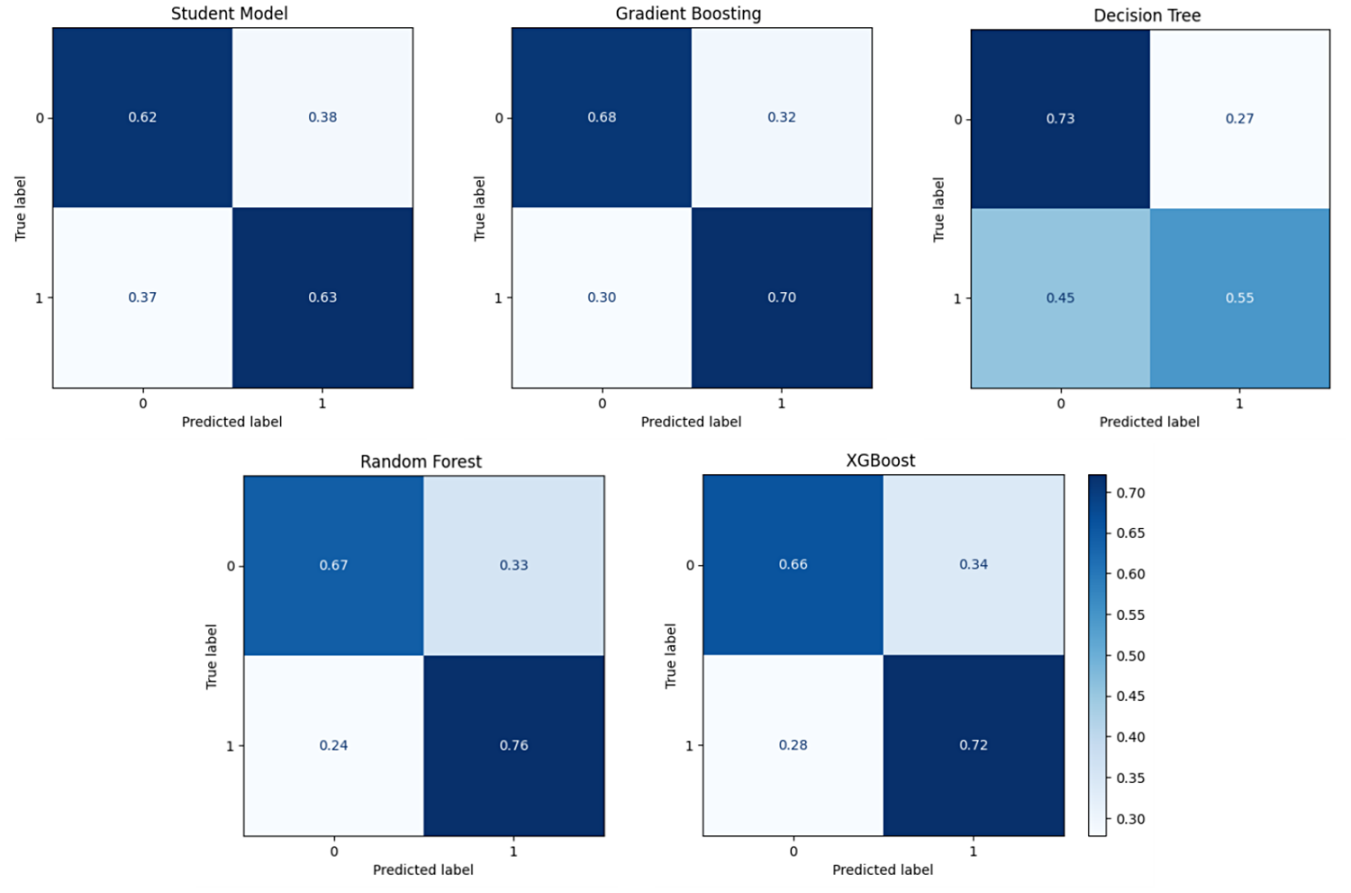
Confusion matrix for experiment 1 showing model predictions on the NIA cohort using the student model trained with knowledge distilled from ADC7

**Fig. 9:**
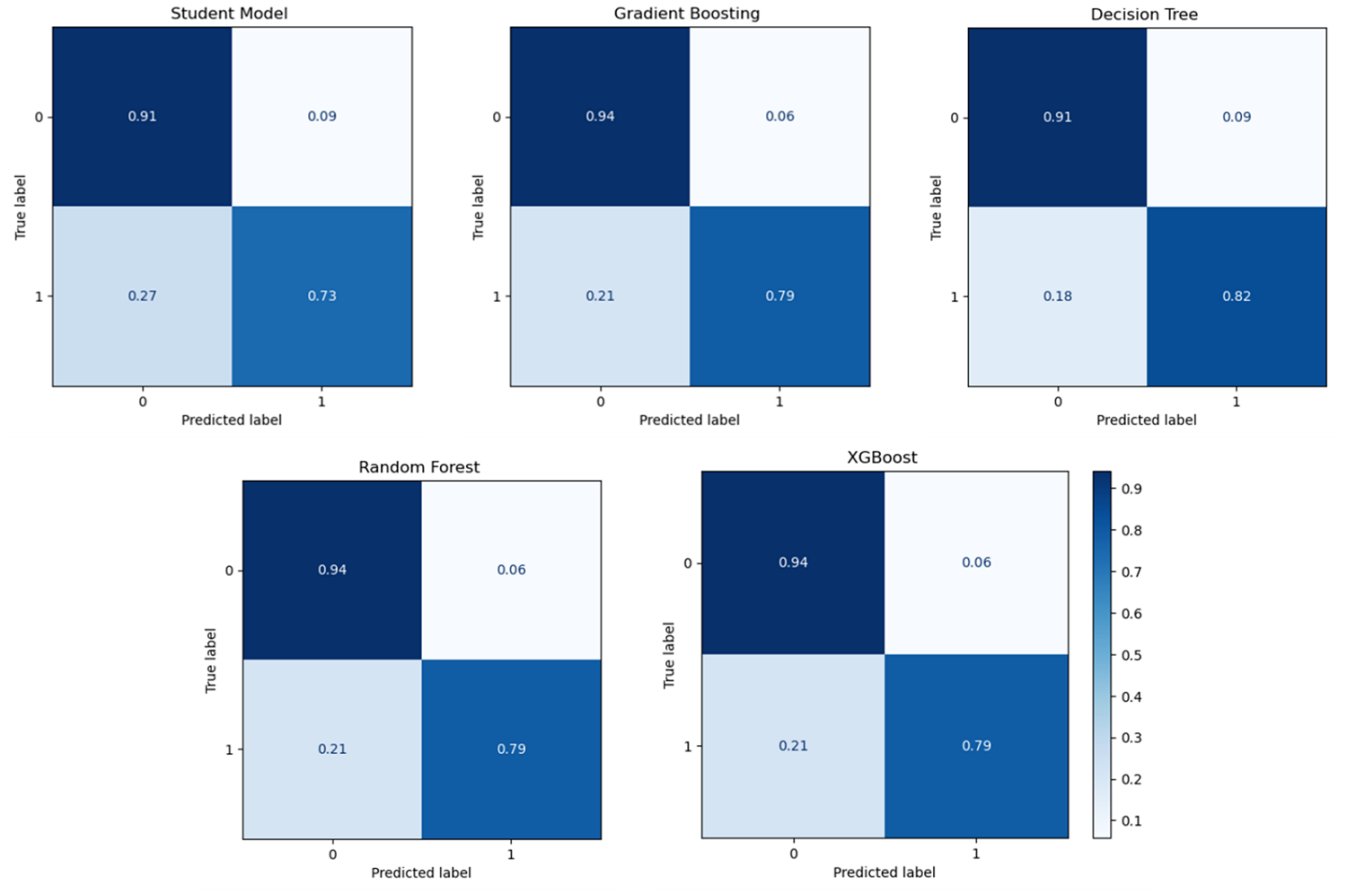
Confusion matrix for experiment 1 showing model predictions on the ROS cohort using the student model trained with knowledge distilled from ADC7

**Fig. 10:**
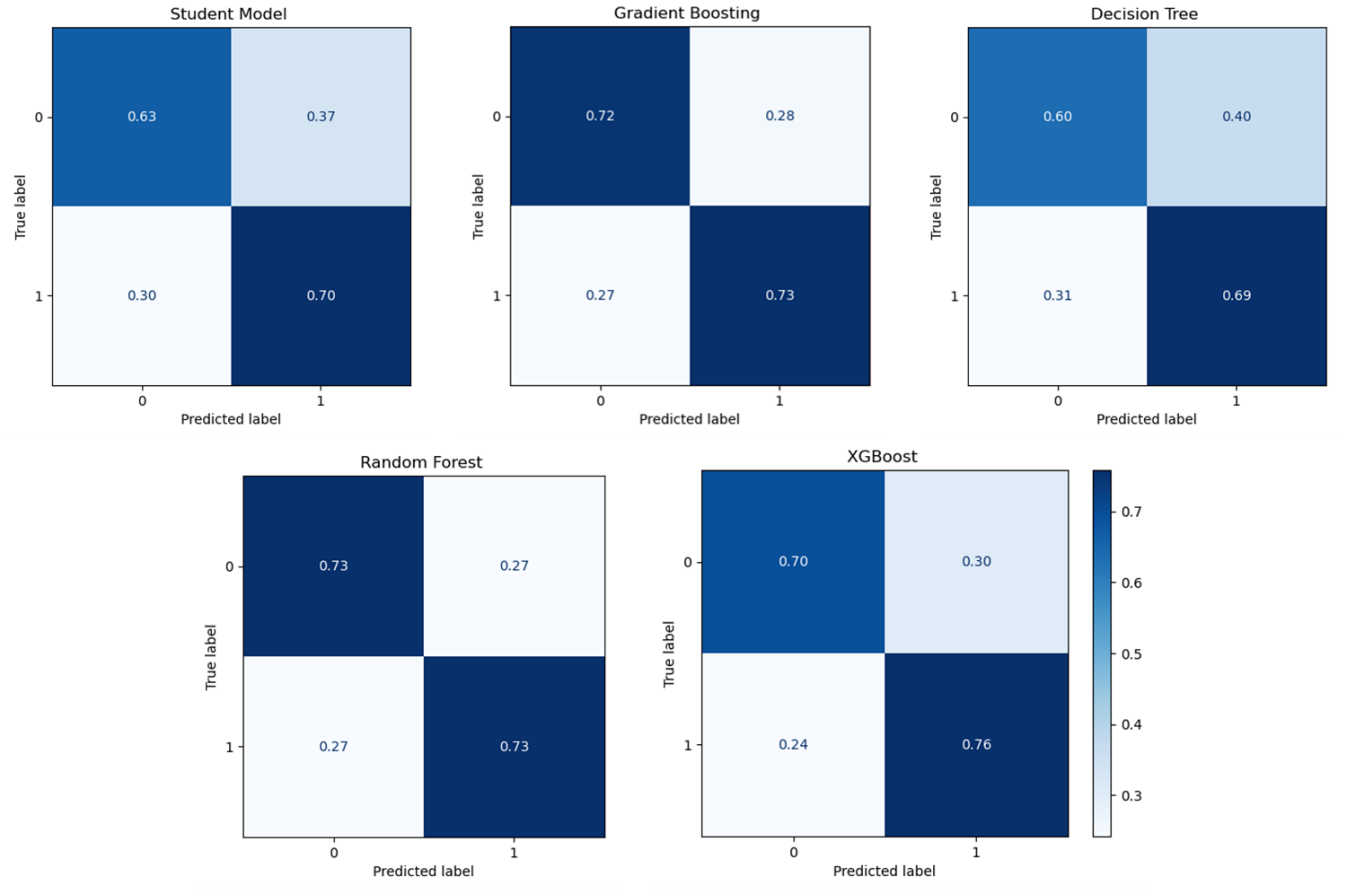
Confusion matrix for experiment 1 showing model predictions on the TGEN cohort using the student model trained with knowledge distilled from ADC7

This experiment investigated the extent to which a model trained on a single source cohort (ADC7) could generalise to target cohorts (BDR, NIA, ROS, and TGEN) without further adaptation. The experiment also compared the performance of the WDNN teacher model, the distilled student model, and traditional classifiers RF, XGBoost, GB, and DT trained on features extracted from the teacher model. All datasets were balanced using combined random oversampling and undersampling to address class imbalance prior to model training. The results, summarised in Table 3, indicate that the distilled student model consistently outperformed the teacher model across all target cohorts, validating the benefit of knowledge distillation. For instance, on the BDR cohort, the student model achieved 65.3% accuracy compared to 54.3% for the teacher. Similar improvements were observed across NIA (63.6% vs. 53.2%), ROS (83.6% vs. 56.0%), and TGEN (68.8% vs. 48.5%). Traditional classifiers trained on the extracted features surpassed both neural models in most cases. RF achieved the highest performance on BDR (84.7%), while XGBoost was most effective for NIA (71.8%) and TGEN (75.1%). The ROS cohort exhibited the highest overall accuracy, with RF reaching 86.6% and DT achieving 85.1%. These findings reflect the strong capability of ensemble tree-based models to generalise across cohorts when equipped with deep feature representations. Figure 6 shows the ROC curves for all models across the four cohorts, reinforcing the superiority of RF and XGBoost in terms of AUC. For example, the RF model on ROS demonstrates a near-perfect AUC, indicating robust discriminative power. The confusion matrices in Figs. 7, 8, 9 and 10 provide further insight into model-specific performance on each target cohort. For the BDR cohort (Fig. 7), tree-based classifiers showed balanced true positive and true negative rates, while the teacher model suffered from misclassification of controls. In contrast, the student model demonstrated improved sensitivity but at the cost of reduced specificity.

**Table 3:** Experiment 1 results for within-cohort transfer learning with a single source dataset (ADC7) is used to train models for a single target dataset. The table compares the performance of the models and the specificity of the teacher-student KD model with traditional classifiers

Source/Target	Model	Acc	Prec	Rec	F1	Spec
Source: ADC7 Target: BDR	Teacher	0.543	0.558	0.560	0.541	0.634
	Student	0.653	0.669	0.653	0.645	0.500
	GB	0.840	0.844	0.840	0.840	0.792
	DT	0.785	0.792	0.785	0.784	0.708
	RF	0.847	0.847	0.847	0.847	0.833
	XGBoost	0.806	0.814	0.806	0.804	0.722
Source: ADC7 Target: NIA	Teacher	0.532	0.550	0.552	0.531	0.634
	Student	0.636	0.645	0.636	0.631	0.758
	GB	0.697	0.697	0.697	0.697	0.703
	DT	0.621	0.624	0.621	0.619	0.691
	RF	0.688	0.689	0.688	0.687	0.649
	XGBoost	0.718	0.718	0.718	0.718	0.703
Source: ADC7 Target: ROS	Teacher	0.560	0.559	0.562	0.553	0.571
	Student	0.836	0.839	0.835	0.835	0.882
	GB	0.806	0.830	0.804	0.802	0.941
	DT	0.851	0.873	0.849	0.848	0.971
	RF	0.866	0.869	0.865	0.865	0.912
	XGBoost	0.791	0.820	0.789	0.785	0.941
Source: ADC7 Target: TGEN	Teacher	0.485	0.480	0.479	0.475	0.455
	Student	0.688	0.692	0.688	0.686	0.618
	GB	0.745	0.747	0.746	0.745	0.702
	DT	0.706	0.711	0.706	0.705	0.634
	RF	0.740	0.740	0.740	0.740	0.728
	XGBoost	0.751	0.753	0.751	0.750	0.707

As shown in Fig. 8 for NIA, the student model performed more conservatively, with moderate false positives and false negatives. Tree-based classifiers, particularly XGBoost and RF, again demonstrated balanced classification. The ROS matrices in Fig. 9 demonstrate excellent performance from all tree-based models, with RF exhibiting minimal error. The TGEN matrices in Fig. 10 show that the student model outperforms the teacher model, although RF and XGBoost remain the most stable in performance.

These results collectively show that while the WDNN architecture with attention is theoretically suited for modelling complex SNP interactions, it underperforms compared to simpler tree-based models in low-sample, low-dimensional tabular settings. Several factors contribute to this trend: (i) the relatively shallow SNP feature space favours ensemble learners; (ii) the neural networks are prone to overfitting, especially in the absence of regularisation or large data; and (iii) traditional models benefit from the distilled, non-linear features generated by the teacher, yielding strong generalisation.

Furthermore, this experiment supports the hypothesis that certain genetic markers are sufficiently conserved across cohorts to enable knowledge transfer. The success of knowledge distillation and teacher-based feature extraction suggests that meaningful patterns learned from ADC7 can be adapted to other datasets, improving cross-cohort AD risk prediction. Moreover, the combination of student model outputs and SHAP-based interpretation presented in Section 4.6 further reinforces the biological plausibility of transferred SNPs, especially those retained in top SHAP-ranked features.

### Exp 2: Cross-cohort - aggregated source $\rightarrow$ single target

**Fig. 11:**
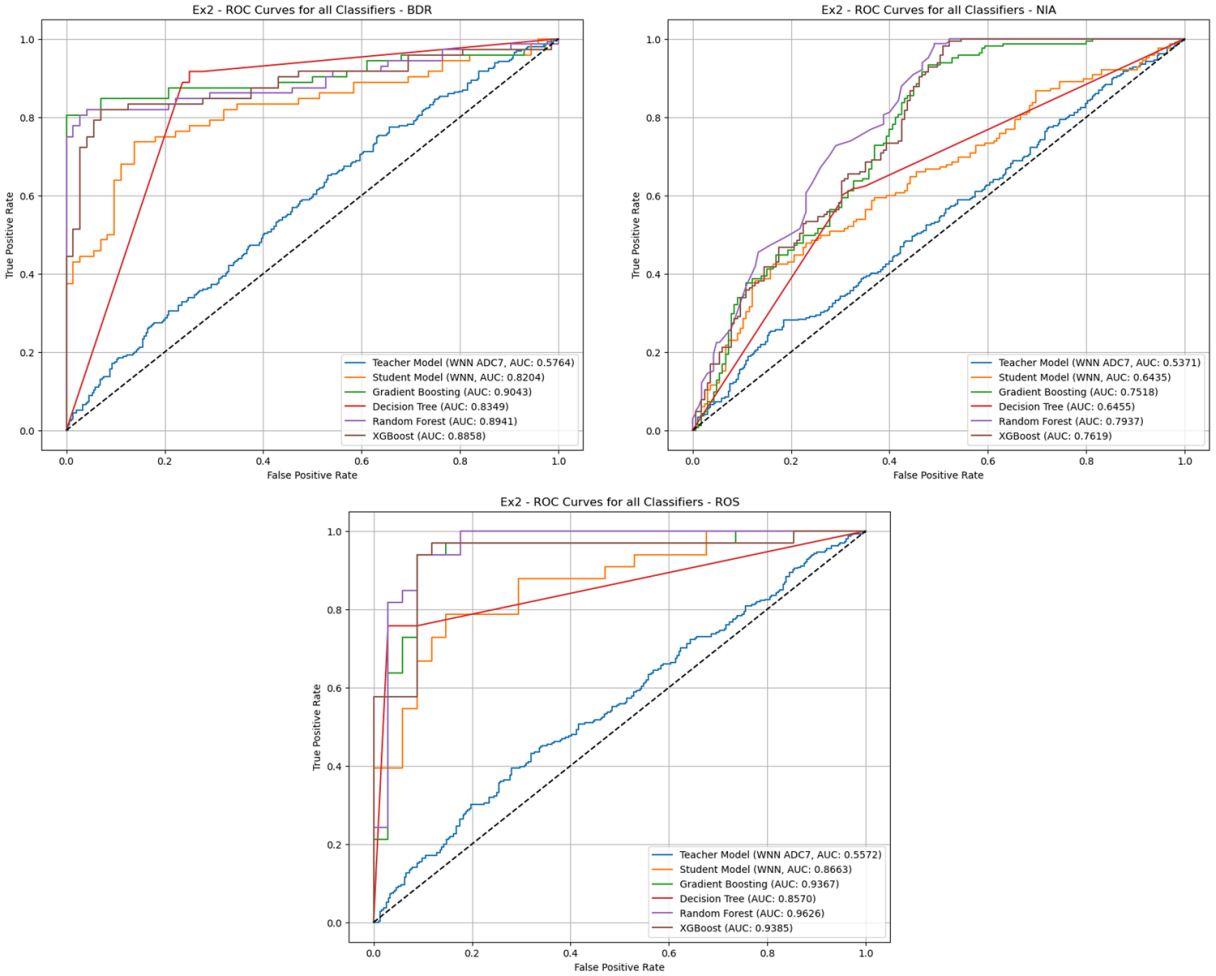
Receiver operating characteristic (ROC) curves for different classifiers in experiment 2, showing performance on BDR, NIA, and ROS target cohorts. The plots compare Teacher/Student models with GB, DT, RF, and XGBoost based on the AUC values

This experiment evaluated the transferability of a model trained on an aggregated dataset (ADC7 + TGEN) to individual target cohorts BDR, NIA, and ROS. The primary objective was to determine whether broader patterns learned from an aggregated dataset could generalise better across diverse cohorts. Table 4 presents the results, providing insights into the relative performance of the models across the target cohorts. DT achieved the highest accuracy at 81.9%, closely followed by GB − 81.3% and RF 80.6% for the BDR cohort. Both DT and GB exhibited strong precision and recall, with DT achieving an F1-score of 81.9% and specificity of 75.0%, indicating very good classification performance. Furthermore, RF demonstrated the highest accuracy at 71.2%, with precision, recall, and F1-score values all aligned at 71.2% for the NIA cohort. This performance underscores RFs ability to generalise well to new cohorts - see also Fig. 11. GB and DT achieved comparable accuracies of 64.6% and 64.5%, respectively. The student model outperformed the teacher model, achieving an accuracy of 59.7% compared to the teacher’s 52.9%. Although the specificity of the teacher model was higher at 76.2%, its overall performance was worse compared to other methods. RF performed best with an accuracy of 89.6%, precision of 89.9%, recall of 89.5%, and an F1-score of 89.5%, coupled with high specificity at 94.1% for the ROS cohort. This was closely followed by XGBoost, which achieved an accuracy of 88.1% and comparable F1-scores. GB and DT also delivered strong performances, with accuracies of 82.1% and 86.6%, respectively.

**Table 4:** Experiment 2 results: cross-cohort - aggregated source to single target. The result compares the performance of teacher/student models with traditional classifiers when trained on ADC7 + TGEN and tested on BDR, NIA, and ROS

Source/Target	Model	Acc	Prec	Rec	F1	Spec
Source: ADC7 + TGEN Target: BDR	Teacher	0.543	0.542	0.542	0.540	0.611
	Student	0.694	0.711	0.694	0.688	0.556
	GB	0.813	0.818	0.813	0.812	0.750
	DT	0.819	0.826	0.819	0.819	0.750
	RF	0.806	0.808	0.806	0.805	0.764
	XGBoost	0.757	0.766	0.757	0.755	0.667
Source: ADC7 + TGEN Target: NIA	Teacher	0.529	0.532	0.525	0.499	0.762
	Student	0.597	0.598	0.597	0.596	0.655
	GB	0.646	0.646	0.646	0.646	0.642
	DT	0.645	0.645	0.645	0.645	0.673
	RF	0.712	0.712	0.712	0.712	0.721
	XGBoost	0.667	0.666	0.666	0.666	0.679
Source: ADC7 + TGEN Target: ROS	Teacher	0.556	0.558	0.554	0.548	0.677
	Student	0.791	0.795	0.790	0.790	0.853
	GB	0.821	0.832	0.820	0.818	0.912
	DT	0.866	0.883	0.864	0.864	0.971
	RF	0.896	0.899	0.895	0.895	0.941
	XGBoost	0.881	0.882	0.880	0.880	0.912

The results of Experiment 2 validate the hypothesis that training on aggregated datasets enhances cross-cohort generalisability. The models benefited from increased diversity, enabling them to generalise better to unseen target cohorts by combining data from multiple source cohorts. The aggregated training dataset enabled models to capture broader patterns across ADC7 and TGEN, improving their generalisability to the target cohorts. Among all models, tree-based algorithms, particularly RF, consistently outperformed others due to their ability to handle high-dimensional SNP data and capture complex non-linear relationships. The strong performance of the student model further demonstrates the utility of KD, where distilled insights allowed the simplified architecture to maintain competitive performance with less computational overhead.

### Exp 3: Cross-cohort - single source $\rightarrow$ aggregated target

Experiment 3 was designed to evaluate the transferability of a model trained on a single GWAS cohort, ADC7, to multiple aggregated target cohorts, such as BDR + NIA, BDR + ROS, and others. The objective was to assess whether training on a single cohort provided sufficient generalisability to predict AD risk across mixed-cohort target sets. The WDNN was benchmarked against machine learning models, including GB, DT, RF, and XGBoost, to compare their performance in cross-cohort settings.

The results shown in Table 5, indicate significant variation in model performance across the different target cohort combinations. In the BDR + NIA target cohort, RF achieved the highest accuracy of 55.5%. The teacher model performed slightly better than GB, DT, and XGBoost but still showed limited generalisability in this context. In the BDR + ROS target cohort, RF and the student model shared the highest accuracy at 68.2%, with the student model showing balanced precision and recall values. RF, while equally accurate, demonstrated slightly higher specificity at 64.4%, suggesting better handling of true negatives. GB and DT performed moderately well, achieving accuracies of 61.9% and 66.5% respectively. The BDR + TGEN target cohort presented a different trend, with RF achieving the highest accuracy of 74.6%. XGBoost followed closely with 73.1%, and GB performed well at 72.5%. The student model performed well with an accuracy of 65.7%, indicating successful knowledge transfer from the teacher model with an accuracy of 49.5%. For the NIA + ROS target cohort, RF again demonstrated the strongest performance, achieving an accuracy of 70.3% and balanced precision, recall, and F1-score values. XGBoost closely followed with an accuracy of 71.8%, while GB and DT achieved 68.3% and 66.8%, respectively. The student model performed well with 65.0% accuracy, significantly outperforming the teacher model, which achieved only 49.2% accuracy. In the NIA + TGEN cohort, XGBoost demonstrated the best performance with an accuracy of 59.3% better than RF, which achieved 57.4%. GB, DT, and the student model followed with accuracies of 54.1%, 55.2%, and 54.2%, respectively. The teacher model showed limited performance, achieving only 51.2% accuracy. ROS + TGEN target cohort, RF performed best achieving an accuracy of 72.2% with a high specificity of 79.0%. XGBoost and GB performed competitively, with accuracies of 66.8% and 67.6%, respectively. The student model achieved 55.4%, outperforming the teacher model’s 52.6%, highlighting the effectiveness of knowledge distillation in transferring insights from a single-source training cohort to mixed-cohort target datasets.

**Table 5:** Experiment 3 results: cross-cohort - single source to multiple targets comparing the performance of teacher/student models and other classifiers when trained on ADC7 and tested on combined target cohorts like BDR + NIA, BDR + ROS, and others

Source/Target	Model	Acc	Prec	Rec	F1	Spec
Source: ADC7 Target: BDR + NIA	Teacher	0.539	0.543	0.545	0.534	0.571
	Student	0.520	0.520	0.520	0.518	0.455
	GB	0.515	0.515	0.515	0.515	0.510
	DT	0.510	0.510	0.510	0.509	0.525
	RF	0.555	0.555	0.555	0.555	0.590
	XGBoost	0.543	0.543	0.543	0.542	0.525
Source: ADC7 Target: BDR + ROS	Teacher	0.550	0.554	0.557	0.545	0.589
	Student	0.682	0.685	0.682	0.681	0.621
	GB	0.619	0.623	0.619	0.616	0.529
	DT	0.665	0.666	0.665	0.664	0.621
	RF	0.682	0.683	0.682	0.682	0.644
	XGBoost	0.642	0.646	0.642	0.640	0.563
Source: ADC7 Target: BDR + TGEN	Teacher	0.495	0.529	0.528	0.495	0.670
	Student	0.657	0.657	0.657	0.656	0.615
	DB	0.725	0.726	0.725	0.725	0.706
	DT	0.681	0.683	0.681	0.681	0.634
	RF	0.746	0.746	0.746	0.746	0.760
	XGBoost	0.731	0.732	0.731	0.731	0.702
Source: ADC7 Target: NIA + ROS	Teacher	0.492	0.503	0.503	0.489	0.554
	Student	0.650	0.650	0.650	0.649	0.688
	GB	0.683	0.683	0.683	0.683	0.683
	DT	0.668	0.671	0.667	0.666	0.744
	RF	0.703	0.703	0.703	0.703	0.709
	XGBoost	0.718	0.718	0.718	0.718	0.739
Source: ADC7 Target: NIA + TGEN	Teacher	0.512	0.498	0.498	0.496	0.438
	Student	0.542	0.542	0.542	0.542	0.538
	GB	0.541	0.541	0.541	0.541	0.548
	DT	0.552	0.552	0.552	0.552	0.522
	RF	0.574	0.574	0.574	0.573	0.555
	XGBoost	0.593	0.593	0.593	0.593	0.591
Source: ADC7 Target: ROS + TGEN	Teacher	0.526	0.512	0.512	0.510	0.455
	Student	0.554	0.554	0.554	0.553	0.576
	GB	0.676	0.676	0.676	0.675	0.649
	DT	0.678	0.681	0.678	0.677	0.610
	RF	0.722	0.726	0.722	0.721	0.790
	XGBoost	0.668	0.670	0.668	0.668	0.620

The results further indicate the performance of tree-based models, particularly RF and XGBoost, consistently outperforming other models across all aggregated target cohorts.

### Exp 4: Fully aggregated transfer (source $\rightarrow$ target)

Experiment 4 evaluates the performance of models trained on aggregated source datasets when applied to aggregated target datasets. The objective was to determine whether training on a more comprehensive dataset, encompassing multiple cohorts, could improve generalisability to other broad datasets. The results of Experiment 4, as shown in Table 6, demonstrate that models trained on aggregated source datasets generally performed well on aggregated targets, with tree-based methods consistently outperforming other models. For the source, a combination of ADC7 + TGEN + BDR applied to target the NIA + ROS, RF achieved the highest accuracy at 71.3%. This performance underscores RFs ability to capture complex relationships within aggregated data. XGBoost and DT followed closely, achieving accuracies of 69.3% and 69.8%, respectively. The student model outperformed the teacher model, achieving 66.5% accuracy compared to the teacher’s 52.4%, reflecting the effectiveness of KD in transferring insights from the aggregated source to the aggregated target. When the ADC7 + TGEN + NIA combination was used as the source and BDR + ROS as the target, GB performed best with an accuracy of 67.1%, narrowly surpassing XGBoost and RF, which achieved accuracies of 64.7% and 63.6%, respectively. While the teacher model struggled with limited generalisability, achieving only 49.8% accuracy, the student model improved significantly, attaining an accuracy of 62.4%.

**Table 6:** Experiment 4 results: fully aggregated target - aggregated source results comparing the performance of teacher/student models and traditional algorithms (GB, DT, RF, XGBoost) using fully aggregated training and testing cohorts

Source/Target	Model	Acc	Prec	Rec	F1	Spec
Source: ADC7 + TGEN + BDR Target: NIA + ROS	Teacher	0.524	0.518	0.517	0.514	0.626
	Student	0.665	0.665	0.665	0.665	0.683
	GB	0.680	0.680	0.680	0.680	0.698
	DT	0.698	0.698	0.698	0.697	0.729
	RF	0.713	0.713	0.713	0.713	0.709
	XGBoost	0.693	0.693	0.693	0.693	0.719
Source: ADC7 + TGEN + NIA Target: BDR + ROS	Teacher	0.498	0.507	0.506	0.486	0.684
	Student	0.624	0.643	0.625	0.613	0.448
	GB	0.671	0.675	0.671	0.669	0.598
	DT	0.619	0.619	0.619	0.618	0.586
	RF	0.636	0.636	0.636	0.636	0.632
	XGBoost	0.647	0.649	0.648	0.647	0.609
Source: BDR + TGEN + ROS Target: ADC7 + NIA	Teacher	0.556	0.527	0.527	0.525	0.668
	Student	0.566	0.566	0.566	0.566	0.546
	GB	0.712	0.712	0.712	0.712	0.720
	DT	0.666	0.668	0.666	0.665	0.720
	RF	0.737	0.740	0.737	0.736	0.676
	XGBoost	0.728	0.728	0.728	0.728	0.734

For the source combination BDR + TGEN + ROS applied to the target ADC7 + NIA dataset, RF again demonstrated the strongest performance with an accuracy of 73.7%, followed by XGBoost at 72.8% and GB at 71.2%. RF and XGBoost consistently outperformed the other models, demonstrating their suitability for transfer learning in aggregated source-to-target scenarios. The high specificity scores achieved by RF and XGBoost, particularly in the ADC7 + TGEN + BDR to NIA + ROS case 70.9% and 71.9%, respectively, indicate their effectiveness in identifying true negatives, further enhancing their reliability.

This experiment validates the hypothesis that training on a more comprehensive dataset improves model generalisability to broad target datasets. Once again, tree-based models, particularly RF, performed better due to their inherent ability to learn from diverse feature distributions and handle imbalanced datasets effectively. While the teacher model showed limited generalisation, the student model consistently improved upon it, demonstrating the KD in simplifying models while maintaining performance.

### Ablation study

**Fig. 12:**
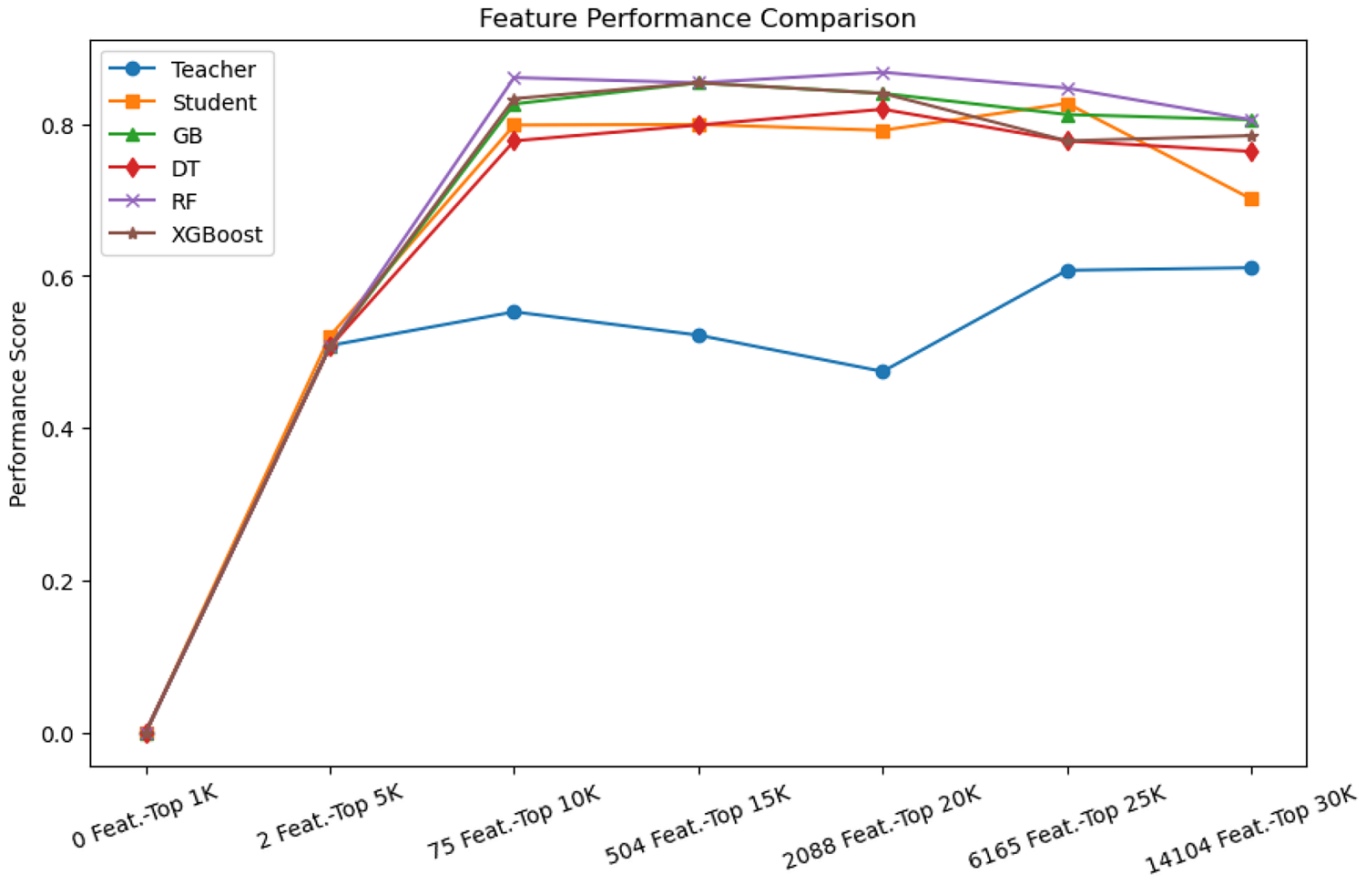
Feature selection analysis showing performance comparison across different subset sizes (5K to 15K features) to evaluate the effectiveness of the process

We conducted ablation experiments to evaluate the effectiveness and influence of key components of our proposed approach, specifically feature selection and the attention mechanism. The influence of feature selection was assessed by evaluating model performance across feature subsets ranging from 1K to 30K, based on feature importance rankings. Results, as illustrated in Fig. 12, indicate that initial increases in feature count improved model performance, highlighting the contribution of relevant genetic markers to AD risk prediction. Given that the 75 most relevant features were consistently present across all cohorts, we utilised this subset in our final experiments to ensure robust and interpretable predictions. The results showed that the optimal number of features was achieved at approximately 10K, where the performance of traditional machine learning models (GB, DT, RF, XGBoost) plateaued. This aligns with our observation that these top 10K features were common across all cohorts, reinforcing their biological relevance in AD risk classification. The teacher-student models exhibited distinct behaviour compared to traditional models.

While traditional models stabilised, the student model showed a slight performance decline at 30K features, suggesting the introduction of noise from less informative markers. The teacher model maintained stable performance, whereas the student model displayed greater variance, reflecting its reliance on knowledge transfer. These findings underscore the importance of feature selection in retaining biologically relevant markers while mitigating the impact of high-dimensional genomic data.

**Fig. 13:**
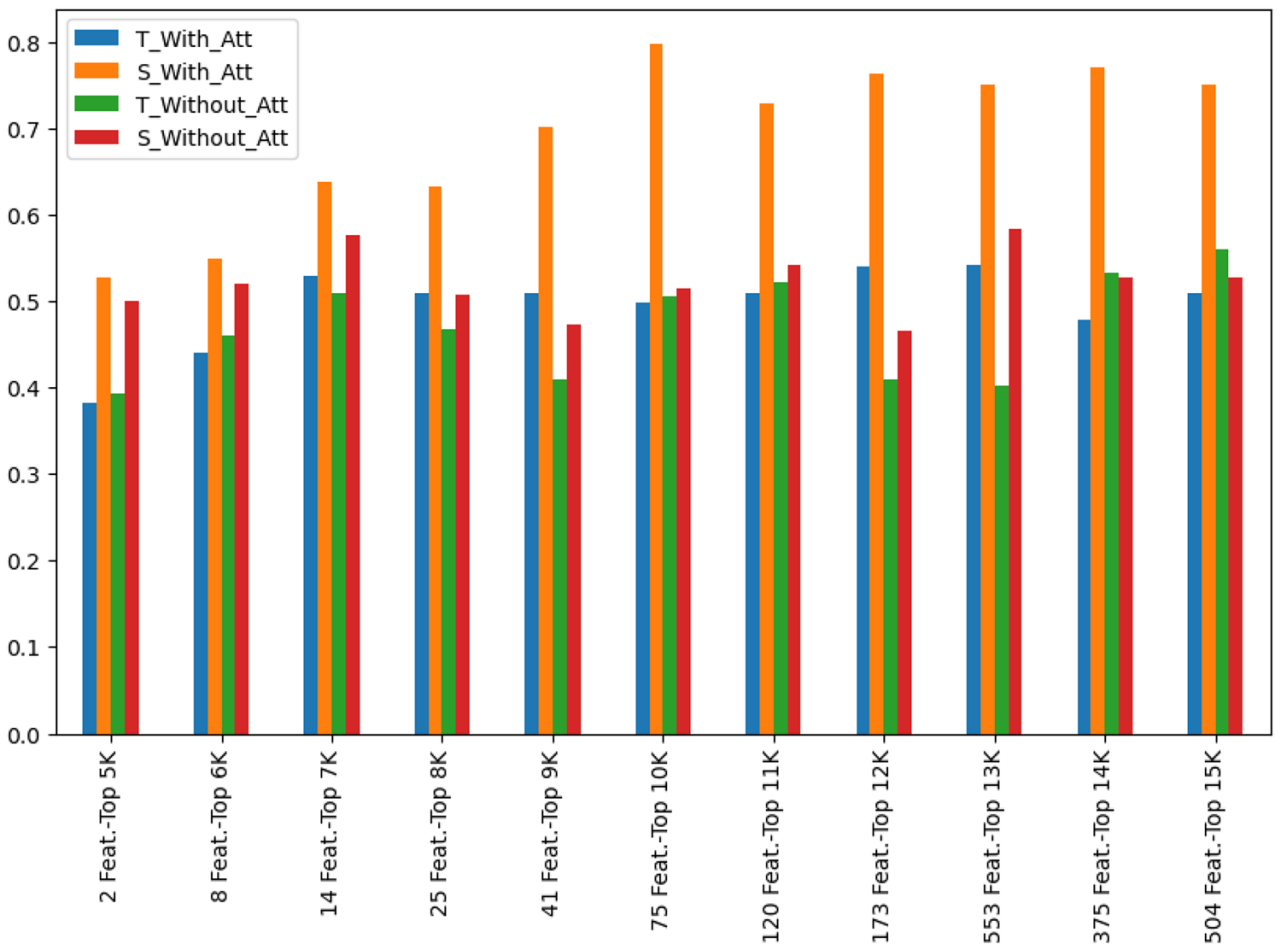
Performance evaluation of Teacher (T) and Student (S) models with and without the MHA mechanism, demonstrating its effectiveness in enhancing KD

Furthermore, we analysed the impact of the MHA mechanism, Teacher (T) and Student (S) models were compared with and without attention (Fig. 13). Models with MHA (T_With_Att, S_With_Att) consistently outperformed those without attention (T_Without_Att, S_Without_Att) across all feature subset sizes. The Student model with attention (S_With_Att) achieved the highest performance, particularly with increasing feature subset sizes, indicating that attention enhances KD by enabling the student model to refine informative patterns. The Teacher model (T_With_Att) showed enhanced feature weighting but maintained stable performance. Models without attention struggled to maintain optimal performance with increasing feature sizes, likely due to their inability to dynamically reweight important genetic features. These findings highlight the crucial role of MHA in improving predictive accuracy, especially within KD frameworks, by enabling selective focus on key genomic features and capturing complex genetic interactions, thereby improving generalisation across diverse cohorts.

### Discussions

The results from Experiments 1–4 offer comprehensive insight into the generalisability, transferability, and efficiency of the proposed cross-cohort KD framework for AD risk prediction using GWAS data. Across all experiments, we evaluated both the direct transferability of neural models and the adaptability of traditional machine learning classifiers trained on distilled deep features. The performance analysis spanning accuracy, F1-score, specificity, and AUC demomstrated both quantitative strengths and interpretability advantages of our hybrid approach.

#### **Generalisability**

The results showed that models trained on a source cohort could generalise to genetically distinct target cohorts, particularly when the source data was diverse or when deep features were used. Tree-based models such as RF and XGBoost consistently outperformed neural architectures, especially in Experiment 1 ADC7 to BDR/NIA/ROS/TGEN. For example, RF achieved 86.6% accuracy in ADC7 to ROS and 74.6% in ADC7 to BDR + TGEN in Experiment 3. These results suggest that ensemble methods are highly effective at utilising distilled features, generalising well across cohorts despite differences in SNP distributions.

#### **Transferability**

The experiments also evaluated the models’ ability to transfer knowledge across varying genetic contexts was further validated in Experiments 2 and 4, where aggregated source cohorts, e.g., ADC7 + TGEN + BDR were used. These models demonstrated enhanced effectiveness and predictiveness when evaluated on heterogeneous target cohorts such as NIA + ROS. For example, RF achieved 73.7% in Experiment 4, compared to 66.5% by the student model. Furthermore, performance gains were not restricted to any one modelling approach but were largely attributable to richer, multi-source training data that better captured inter-cohort genetic variability.

#### **Efficiency**

The student model, trained via KD, was consistently more efficient than the teacher model, requiring fewer parameters and training resources while achieving comparable or improved accuracy. In Experiment 2 ADC7 + TGEN to BDR, the student achieved 69.4%, outperforming the teacher’s model 54.3%. These results confirm that the distilled model successfully retained the most salient knowledge from the teacher, enabling lightweight deployment in computationally constrained environments, an advantage also reported in other biomedical KD applications [29].

#### **Model performance**

Tree-based models, particularly RF and XGBoost, consistently outperformed other methods in terms of accuracy, F1-score, precision, recall, and specificity. Their adaptability and robustness across all experimental setups highlight their suitability for transfer learning tasks in GWAS data. For example, RF achieved high specificity scores across Experiments 2–4, ensuring reliable identification of true negatives, which is critical in medical predictions.

#### **Interpretability with SHAP**

**Fig. 14:**
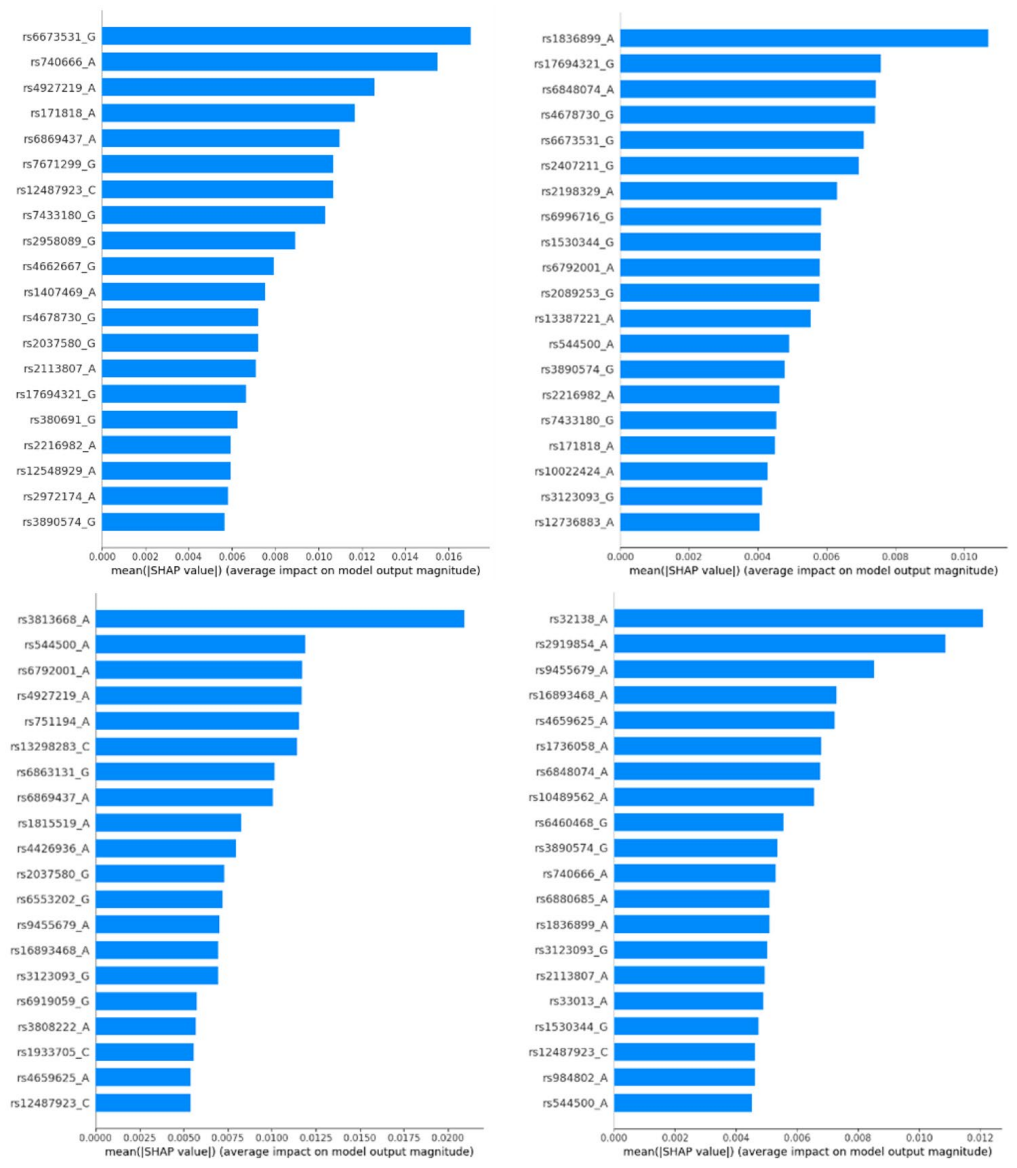
SHAP summary plots showing the top 20 SNPs ranked by impact on the output of the student model across each target cohort. The teacher model was trained on ADC7, and knowledge was transferred to four target cohorts: BDR (top left), NIA (top right), ROS (bottom left), and TGEN (bottom right). Variation in ranked SNPs across cohorts reflects biological and data-driven differences in feature attribution under the knowledge distillation framework

To investigate the biological plausibility of our models and gain insight into knowledge transfer, we applied SHAP interpretability analysis exclusively on the student models (Fig. 14). These models embody the distilled knowledge from ADC7 and adapt it to the unique genotype distributions of BDR, NIA, ROS, and TGEN.

SHAP results revealed that certain biologically relevant SNPs, such as rs544500_A, associated with the ABI3BP gene known for its role in extracellular matrix composition and immune response regulation and rs740666_A, linked to the SYN3 gene, which encodes Synapsin III, a key regulator of synaptic plasticity and vesicle trafficking implicated in AD, were consistently ranked highly across multiple cohorts. These consistent patterns support the transferability of biologically meaningful signals through the proposed KD approach. In contrast, some high-impact SNPs like rs429358, widely known for its strong association with AD, were sometimes positioned outside the ranking of top features and SHAP thresholds, suggesting that model-specific attribution can diverge from traditional GWAS significance rankings, particularly in the presence of complex feature interactions.

#### **Biological relevance and novelty**

Table 7 presents a curated list of SNPs from SHAP analysis, enriched with known or hypothesised relevance to AD biology. While genes like GRM7 and FCGR3A have established links to neuroinflammation and glutamatergic signalling, other variants such as rs3813668_A (LINC02997) show no known annotation suggesting the potential discovery of novel cohort-specific associations. Specifically, no SNP appeared universally in all four cohorts, implying that each student model reinterprets ADC7 knowledge based on local allele distributions a key insight only made possible through SHAP-based analysis.

**Table 7:** Top SHAP-ranked SNPs with biological relevance and cross-cohort presence

rsID	Chromosome	Gene(s)	Biological Relevance	Cohorts Present In
rs6673531_G	NC_000001.11	None	-	BDR, NIA
rs740666_A	NC_000005.10	SYN3	Encodes Synapsin III, involved in synaptic function; variants linked to AD risk via effects on synaptic plasticity and vesicle trafficking	BDR, TGEN
rs4927219_A	NC_000001.11	MIR4422HG	Gene locus associated with AD and cognitive decline; direct SNP association limited	BDR, ROS
rs1836899_A	NC_000004.12	FCGR3A	Encodes Fc*γ*RIIIa receptor involved in IgG-mediated immune responses; variations influence AD susceptibility via neuroinflammation	NIA, TGEN
rs17694321_G	NC_000003.12	GRM7	Involved in glutamate signalling pathways that are linked to and known to be dysregulated in AD.	BDR, NIA
rs6848074_A	NC_000004.12	LINC02261	Associated with NPY-LA levels linked to immune regulation and neurovascular pathways	NIA, TGEN
rs3813668_A	NC_000005.10	LINC02997	No established biological relevance to AD	ROS
rs544500_A	NC_000003.12	ABI3BP	Gene involved in extracellular matrix and immune processes	NIA, ROS, TGEN
rs6792001_A	NC_000003.12	ACE	Intronic variant in ACE; gene linked to AD via roles in amyloid-beta clearance and vascular pathways	NIA, ROS
rs32138	NC_000005.10	CTNND2	Encodes delta-catenin, involved in synaptic function and neuronal development and linked to AD-related neurodegeneration.	TGEN
rs2919854_A	NC_000002.12	SMYD1	With transcription factor skNAC, regulates genes implicated in AD pathology, including Tau; loss affects neurodegenerative gene networks	TGEN
rs9455679_A	NC_000006.12	SMOC2	Modulates microglial activity and amyloid-beta pathology; elevated in AD and a potential biomarker and therapeutic target	TGEN

#### **Benchmarking against existing literature**

As shown in Table 8, our models outperform several state-of-the-art approaches in GWAS-based AD prediction. Unlike previous studies that primarily focused on within-cohort classification using larger SNP sets, this study introduces a cross-cohort framework that achieves strong generalisability with a reduced set of only 75 SNPs. For example, RF trained on teacher-extracted features achieved 86.6% and 89.9% accuracy in Exp 1 and 2, respectively, outperforming earlier ensemble approaches and matching or exceeding the performance of single-cohort WDNNs. This was made possible through the integration of a KD pipeline, where a wide and deep teacher model transfers learned representations to a compact student model, and feature extraction is applied to enhance traditional classifiers. In addition, by incorporating SHAP for post hoc interpretation, we connect predictive performance with biological relevance, an aspect often missing in earlier models. These methodological differences contribute to both the improved accuracy and the interpretability observed across experiments, demonstrating the robustness and practical value of our dual-path transfer learning approach across heterogeneous genomic datasets.

**Table 8:** Performance comparison with existing literature on GWAS-Based ad prediction

Study	Model	Acc.	Features	Dataset
Oriol et al. [37]	Ensemble Models	~70%	2500	ADNI
Segura et al. [38]	Gradient Boosted Trees	80%	145	ADNI3
Taeho et al. [39]	CNN	75%	4000	ADNI
Alatrany et al. [22]	WNN (EXP2)	99%	747	ADNI
Alatrany et al. [22]	WDNN (EXP4)	83%	121 (W) + 4697 (D)	ADNI
**This study**	RF on WDNN features (EXP1)	**86.6%**	75	ADC7 $\rightarrow$ ROS
**This study**	RF on WDNN features (EXP2)	**89.9%**	75	ADC7 + TGEN $\rightarrow$ BDR

## Limitations, ethical considerations, and practical implications

While this study demonstrates encouraging results for cross-cohort GWAS-based AD risk prediction, several limitations and considerations must be acknowledged to guide future research and ensure responsible deployment.

### **Limitations**

A major limitation lies in the variability of GWAS datasets across cohorts. Differences in genotype imputation strategies, sample sizes, population structures, and quality control pipelines introduce heterogeneity that may affect model generalisability. Although TL and KD helped mitigate these effects, performance may still degrade when applied to cohorts that are demographically or genetically underrepresented. In particular, the proposed models were evaluated on cohorts primarily composed of individuals of European ancestry, limiting insights into cross-ancestry robustness. Furthermore, while the SHAP-based interpretability component enabled post hoc analysis of feature attribution, it was applied only to the student models. Broader application of interpretability tools across all model components could uncover deeper insights into the pathways of knowledge transfer and feature reweighting.

### **Ethical considerations**

The application of machine learning to genomic data raises important ethical challenges. Privacy concerns surrounding patient genetic information necessitate strict adherence to data protection regulations, such as the General Data Protection Regulation (GDPR) [40] and the Health Insurance Portability and Accountability Act (HIPAA) [41]. The potential misuse of genetic risk scores particularly in contexts such as insurance or employment raises the spectre of genetic discrimination if safeguards are not properly enforced. Although ancestry was not explicitly modelled in this study, we recognise the limitations of current public GWAS datasets in representing non-European populations. Future work must address these gaps through the inclusion of more diverse, ancestry-aware cohorts and the integration of fairness-aware learning techniques to ensure equity in predictive outcomes.

### **Practical implications**

Despite these limitations, the framework introduced here has substantial practical value for advancing AD risk stratification and personalised medicine. By improving SNP selection through F-score-based filtering and using feature-rich WDNN, the models offer a biologically grounded and interpretable approach to genetic risk prediction. The use of KD and feature extraction pathways enables the development of lightweight, generalisable models that retain predictive performance while reducing computational costs. These qualities are particularly important for applications in clinical or resource-limited environments. From a translational perspective, effective implementation of such models in real-world settings will require interdisciplinary collaboration between geneticists, clinicians, and computational scientists. While AI-driven genomic models offer considerable promise, clinical validation remains essential before deployment in diagnostic workflows.

## Conclusion

This paper presented an approach for cross-cohort AD risk prediction using GWAS data, utilising a novel combination of GWAS, KD, traditional classifiers, and post hoc SHAP-based interpretability. By systematically evaluating four TL scenarios ranging from single-cohort to fully aggregated multi-cohort experiments, this work demonstrated the effectiveness, adaptability, and interpretability of the proposed dual-path strategy for generalisable AD prediction.

The proposed KD-based teacher–student architecture significantly improved model efficiency without compromising accuracy. Across all experiments, student models consistently outperformed the teacher models, affirming KDs ability to retain biologically informative representations in a computationally lightweight form. Tree-based classifiers including, RF and XGBoost trained on deep features extracted from the teacher model achieved superior accuracy, underscoring the utility of deep feature transfer even for traditional models. In particular, RF achieved an accuracy of 89.9% in cross-cohort evaluation (Exp 2), outperforming several state-of-the-art methods while using a compact SNP feature set. Training on aggregated cohorts significantly enhanced model transferability by providing more diverse genetic representations, especially for heterogeneous target populations. These results underscore the critical role of genetic diversity in developing inclusive and generalisable predictive models, a key factor for equitable precision medicine.

A critical aspect of this work lies in its integration of SHAP interpretability with KD. We were able to demonstrate how knowledge distilled from ADC7 was differentially interpreted across cohorts by applying SHAP to student models. SNPs such as rs544500_A (ABI3BP) and rs740666_A (SYN3) were consistently ranked across multiple target datasets, indicating preserved biological relevance. Interestingly, SNPs such as rs429358 did not consistently appear among the top-ranked features based on SHAP thresholds. This indicates a model-specific reinterpretation of their importance and emphasises the role of interpretability in uncovering subtle attribution patterns. Ultimately, this biologically informed analysis enhances the KD utility by improving its transparency and trustworthiness, moving beyond just predictive accuracy

In conclusion, this paper delivers a novel, interpretable, and scalable approach to AD risk prediction. It bridges deep learning and classical ML via knowledge transfer and offers biological insight through SHAP, providing a framework that is both technically robust and biologically meaningful. These results establish a clear advancement over previous studies, which have largely focused on within-cohort learning or lacked interpretability.

To build on these findings, future research should explore domain adaptation strategies such as adversarial and contrastive representation learning to further improve cross-population transferability. Incorporating multi-omics data with lifestyle and environmental factors, could enrich risk stratification. Moreover, extending this approach to longitudinal GWAS datasets may enable early detection and modelling of AD progression, potentially supporting preclinical intervention. Bridging computational advances with clinical validation remains essential for real-world deployment, requiring closer integration with diagnostic pipelines and cross-disciplinary collaboration.

## Data Availability

The genotypes for the Brains for Dementia Research BDR Cohort dataset are freely available via the https://www.dementiasplatform.uk/. The Alzheimer’s Disease Centres seventh set of ADC genotyped subjects used by the Alzheimer’s Disease Genetics Consortium (ADGC) ADC7 (NG00071), the National Institute on Aging Genetics Initiative for Late-Onset Alzheimer’s Disease (NIA-LOAD), NIA (NG00020), TGen II, TGEN (NG00028), and the Religious Orders Study and Memory and Aging Project, ROSMAP can be downloaded from https://www.niagads.org/. The code implementation for the model is publicly available on GitHub and can be accessed at: https://github.com/isibor-ihianle/AD-Risk-Transfer-Learning.

## Footnotes

1 https://www.dementiasplatform.uk/

2 https://www.niagads.org/
